# Detection and Molecular Characterization of Two *FAD3* Genes Controlling Linolenic Acid Content and Development of Allele-Specific Markers in Yellow Mustard (*Sinapis alba*)

**DOI:** 10.1371/journal.pone.0097430

**Published:** 2014-05-13

**Authors:** Entang Tian, Fangqin Zeng, Kimberly MacKay, Vicky Roslinsky, Bifang Cheng

**Affiliations:** Agriculture and Agri-Food Canada, Saskatoon Research Centre, Saskatoon, SK, Canada; National Key Laboratory of Crop Genetic Improvement, China

## Abstract

Development of yellow mustard (*Sinapis alba* L.) with superior quality traits (low erucic and linolenic acid contents, and low glucosinolate content) can make this species as a potential oilseed crop. We have recently isolated three inbred lines Y1127, Y514 and Y1035 with low (3.8%), medium (12.3%) and high (20.8%) linolenic acid (C18∶3) content, respectively, in this species. Inheritance studies detected two fatty acid desaturase 3 (*FAD3*) gene loci controlling the variation of C18∶3 content. QTL mapping revealed that the two *FAD3* gene loci responsible for 73.0% and 23.4% of the total variation and were located on the linkage groups Sal02 and Sal10, respectively. The *FAD3* gene on Sal02 was referred to as *SalFAD3.LA1* and that on Sal10 as *SalFAD3.LA2*. The dominant and recessive alleles were designated as *LA^1^* and *la^1^* for *SalFAD3.LA1*, and *LA^2^* and *la^2^* for *SalFAD3.LA2*. Cloning and alignment of the coding and genomic DNA sequences revealed that the *SalFAD3.LA1* and *SalFAD3.LA2* genes each contained 8 exons and 7 introns. *LA^1^* had a coding DNA sequence (CDS) of 1143 bp encoding a polypeptide of 380 amino acids, whereas *la^1^* was a loss-of-function allele due to an insertion of 584 bp in exon 3. Both *LA^2^* and *la^2^* had a CDS of 1152 bp encoding a polypeptide of 383 amino acids. Allele-specific markers for *LA^1^*, *la^1^*, *LA^2^* and *la^2^* co-segregated with the C18∶3 content in the F_2_ populations and will be useful for improving fatty acid composition through marker assisted selection in yellow mustard breeding.

## Introduction

Yellow mustard (*Sinapis alba* L., 2*n* = 24) is cultivated as an important condiment crop. It has many desirable agronomic traits such as resistance to cabbage aphids [1], flea beetles [2], [3] and blackleg diseases [4]. In addition, it is drought tolerant and resistant to pod shattering. Yellow mustard germplasm with canola quality (low erucic acid and low glucosinolate contents) was developed at Agriculture and Agri-Food Canada-Saskatoon Research Centre (AAFC-SRC) [5], which makes yellow mustard have the potential to become an alternative oilseed crop to canola *B. napus*, especially in semi-arid areas.

The oil quality of canola *B. napus* is determined by the proportion of the three major unsaturated fatty acids: oleic acid (C18∶1), linoleic acid (C18∶2) and linolenic acid (C18∶3). Traditional *B. napus* cultivars contain 9% C18∶3 of the total fatty acids [6]. The high level of linolenic acid in canola oil is undesirable since it shortens the shelf life and causes off-type flavour of the oil due to the three easily oxidized double bonds. A low linolenic acid mutant, containing 3–5% C18∶3, was produced by ethyl methanesulfonate (EMS) treatment of a high C18∶3 *B. napus* cv. Oro seed [7]. Current low C18∶3 canola cultivars have been developed using this low linolenic gene source.

Linolenic acid content is determined mainly by the embryonic genotype with some influence from temperature, maternal genotype and cytoplasm in *B. napus* [8]–[10]. QTL mapping identified two major QTLs, accounting for 25.2–28.8% and 52.4–62.7% of the C18∶3 variation, located on the linkage groups A4 and C4, respectively, in *B. napus* [11], [12]. It was reported that the low C18∶3 variant resulted from mutations of *FAD3* genes in *B. napus* [11]–[13]. The *FAD3* gene on A4 harboured a C to T substitution in exon 7, which when translated causes the wild type amino acid arginine to be replaced by cysteine. The *FAD3* gene on C4 contained a G to A substitution in the 5′ splice site of intron 6 in the low C18∶3 *B. napus* line. *FAD3* allele-specific markers based on the sequence variation were developed and proved to be useful for identification of different C18∶3 genotypes in canola *B. napus* [11], [12]. Yellow mustard accessions contain 6.9–12.4% linolenic acid of total fatty acids in the seed [14], [15]. Recently, inbred lines with high (18.5%), medium (13.8%) and low (3.8%) linolenic acid content, respectively, have been obtained through inbreeding of heterozygous open-pollinated plants in yellow mustard [16].

The low linolenic acid variant (3.8%) is a valuable gene source for breeding canola-quality yellow mustard with high stability oil (high oleic and low linolenic acids) as that of canola *B. napus*. The knowledge about genetic and molecular bases of the variation in C18∶3 content and development of *FAD3* allele-specific markers will greatly facilitate the development of low linolenic canola-quality yellow mustard. The objectives of this study were: 1) to determine the inheritance and perform QTL mapping of the C18∶3 content; and 2) to clone the *FAD3* genes and further develop allele-specific markers for marker assisted selection.

## Materials and Methods

### Plant Materials

Linolenic acid contents of the three parental lines Y1127, Y514 and Y1035 are shown in Table 1. Y1127 is an S_4_ inbred line produced by selfing of the low linolenic S_2_ line Y158 for two generations and has a low C18∶3 content (average: 3.8%). Y514 is the doubled haploid line SaMD3 [17] and has a medium C18∶3 content (average: 12.3%). Y1035 is an S_4_ inbred line and has a high C18∶3 content (average: 20.8%).

**Table 1:** Linolenic acid contents of the parental lines Y1127, Y514, Y1035 and F_1_ seeds, and the mid-parental value in yellow mustard.

Genotype	Generation	Linolenic Acid Content* (% of total fatty acids)
Y1127	S_4_	3.8±0.7
Y514	DH	12.3±0.7
Y1035	S_4_	20.8±0.8
Y1127×Y1035	F_1_	13.7±1.3
Mid parent value		12.3
Y1127×Y514	F_1_	8.9±0.7
Mid parent value		8.0
Y514×Y1035	F_1_	15.3±0.7
Mid parent value		16.5

*: Linolenic acid content is expressed as mean value ± standard deviation.

The F_1_ seeds of the three crosses Y1127 (low)×Y1035 (high), Y1127 (low)×Y514 (medium) and Y514 (medium)×Y1035 (high) were produced. To produce the BC_1_ seeds, the F_1_ plants of the three crosses were crossed as the female with the parental line with a lower C18∶3 content. All plants were raised under the same conditions in the greenhouse at AAFC-SRC.

### Regional Linkage Mapping

Regional linkage mapping of the linolenic acid content was performed using intron length polymorphism (ILP) markers and bulked segregant analysis (BSA) [18]. A total of 1478 ILP primer pairs: 380 from *Arabidopsis thaliana* [19] and 1098 from *B. napus* [20] were used to screen the three parental lines for polymorphic markers. The high bulk was made by mixing equal amount of DNA from 10 F_2_ plants with the highest C18∶3 content, while the low bulk was formed from 10 F_2_ plants with the lowest C18∶3 content for each of the three crosses. The primers detecting polymorphic markers between the two bulks were subsequently used to genotype individual plants of the three F_2_ populations. Genomic DNA was extracted from young leaves of the parental lines Y1127, Y514 and Y1035, F_1_ and F_2_ plants using a modified sodium dodecyl sulfate method [21]. Each PCR (20 µl) contained 1× standard PCR buffer (NEB), 1 U of Taq polymerase (NEB), 0.25 µM forward primer, 0.25 µM reverse primer, 100 µM each dNTP and 50 ng of genomic DNA in a total volume 20 µL. The PCR amplification consisted of an initial denaturation at 94°C for 5 min, 35 cycles consisting of 94°C (45 sec), 55°C (45 sec), 72°C (1 min) terminating with 72°C for 7 min. All PCR products were analyzed by electrophoresis in 2% agarose gels in 1× Tris-acetate-ethylenediaminetetraacetic acid buffer. Gels were visualized by staining in ethidium bromide and photographed on a digital gel documentation system.

The regional linkage map of C18∶3 content was constructed using JoinMap 4.0 [22] with a minimum LOD threshold of 4.0. QTL analysis of C18∶3 content was performed using the interval mapping method of MapQTL 6.0 [23]. A Chi-square test was used for evaluating the genetic model of C18∶3 content in the BC_1_ and F_2_ populations, and the ILP markers in the F_2_ populations.

### Cloning of the Coding Region of the *FAD3* Gene

Primer pair No 1 (Table S1) was designed based on the conserved coding regions of the *FAD3* genes in *B. napus* and *A. thaliana.* It was used to clone the coding DNA sequence (CDS) of the *FAD3* gene in yellow mustard. Immature seeds at 22 days after pollination were collected from two individual plants from each of the parental lines. Total RNA was extracted from the immature seeds using the RNeasy Plant Mini Kit (Qiagen) as per the manufacturer’s instructions. 750 ng of RNA from each of the parental lines was used to prepare the cDNA using Qiagen’s Omniscript RT Kit as per the manufacturer’s instructions. Each PCR (20 µl) contained 1× PCR standard buffer (NEB), 100 µM of each dNTP, 0.25 µM of each forward and reverse primer, 1 U of Taq polymerase (NEB) and 50 ng of cDNA. Polymerase chain reaction was performed with an initial denaturation at 94°C for 3 min followed by 35 cycles of 45 s at 94°C, 30 s at 55°C and 1 min at 72°C with a final extension cycle of 72°C for 10 min.

### Cloning of the 5′ and 3′ Flanking Sequences and the Genomic DNA Sequences of the *FAD3* Genes

Primer pairs No 2 and 3 (Table S1) were designed based on the 5′ coding sequences of the cloned *SalFAD3.LA1* and *SalFAD3.LA2* genes, respectively. They were used to clone the 5′ upstream sequences by PCR walking according to the protocol of Siebert et al. [24]. Primer No 4 (Table S1) was designed based on the 3′ coding sequences of the cloned *SalFAD3.LA1* and *SalFAD3.LA2* genes, and was used to clone the 3′ flanking sequence by PCR walking. Primer pairs No 5 and 6 (Table S1) were designed based on the 5′ flanking sequence and the 3′ flanking sequence of the cloned *SalFAD3.LA1* and *SalFAD3.LA2* genes, respectively, and were used to clone the genomic DNA sequences of *SalFAD3.LA1* and *SalFAD3.LA2* genes. The standard protocol from the Clontech kit (website: www.clontech.com, Protocol PT 3042, Version PR 03300) by Gwyneth Ingram and Karine Coenen was followed to facilitate the PCR walking.

### DNA Sequencing

The expected PCR bands were cloned using the pGEM-T Vector System I (Promega) following the provided instructions. The plasmids were extracted using the QiaSpin Kit (Qiagen) following the manufacturer’s instructions and sequenced using the primer pairs No 7–11 (Table S1) at the Plant Biotechnology Institute, National Research Council, Canada.

### Phylogenetic Tree

The multiple alignments were performed using ClustalW (http://www.ebi.ac.uk/clustalw/). MEGA software (version 4.0) (http://www.megasoftware.net/index.html)[25] was used to construct a phylogenetic tree with the aligned protein sequences. The neighbor-joining method was used with the pairwise deletion option, poisson correction model, and the 1000 bootstrap replicates test.

### Development of the *SalFAD3.LA1* and *SalFAD3.LA2* Allele-specific Markers

The *SalFAD3.LA1* and *SalFAD3.LA2* allele-specific markers were generated using primer pair No 12 (Table S1) which was designed based on the conserved flanking sequences of intron 3. The PCR reaction was performed with LongAmp Taq 2× Master Mix (NEB) following the manufacturer’s instructions with a 60°C annealing temperature.

### Fatty Acid Analysis

Seed fatty acid composition was analyzed according to [26] with the following modification: the gas chromatography of the methyl esters was performed with a HP-INNOWax fused silica capillary column (0.25 mm by 0.5 m and 7.5 µm) (Agilent Technologies) at 250°C using hydrogen as the carrier gas. A minimum of 10 seeds from each of the parental lines and F_1_ hybrids as well as 160 F_2_ seeds of each of the three crosses were half-seed analyzed according to [27]. Ninety-six seeds from each of the BC_1_ populations were analyzed using the single seed method.

## Results

### Linolenic Acid Content is Controlled by Two Gene Loci in Yellow Mustard

**Figure 1:**
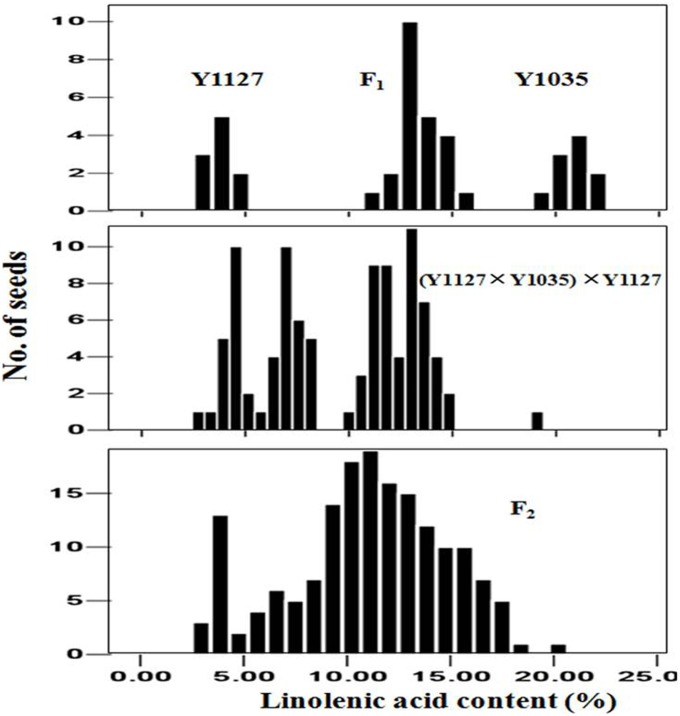
Frequency distributions of linolenic acid contents in individual seeds of Y1127, Y1035, F_1_, (Y1127×Y1035)×Y1035 and F_2_ populations.

**Figure 2:**
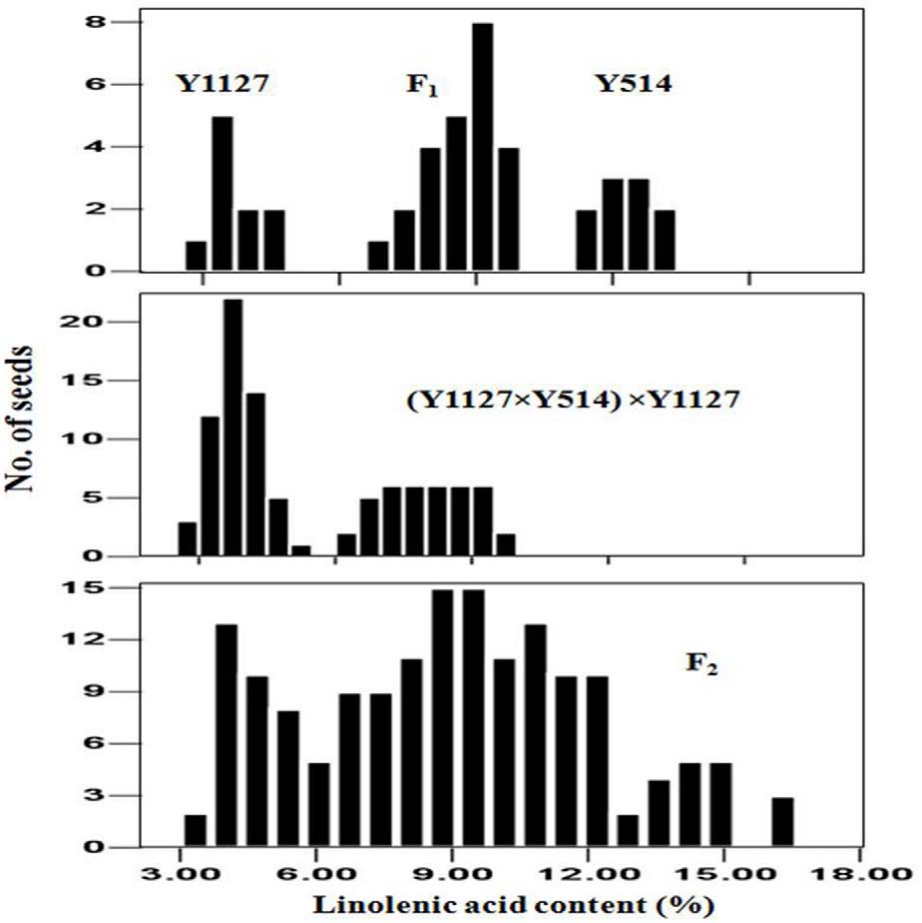
Frequency distributions of linolenic acid contents in individual seeds of Y1127, Y514, F_1_, (Y1127×Y514)×Y1127 and F_2_ populations.

**Figure 3:**
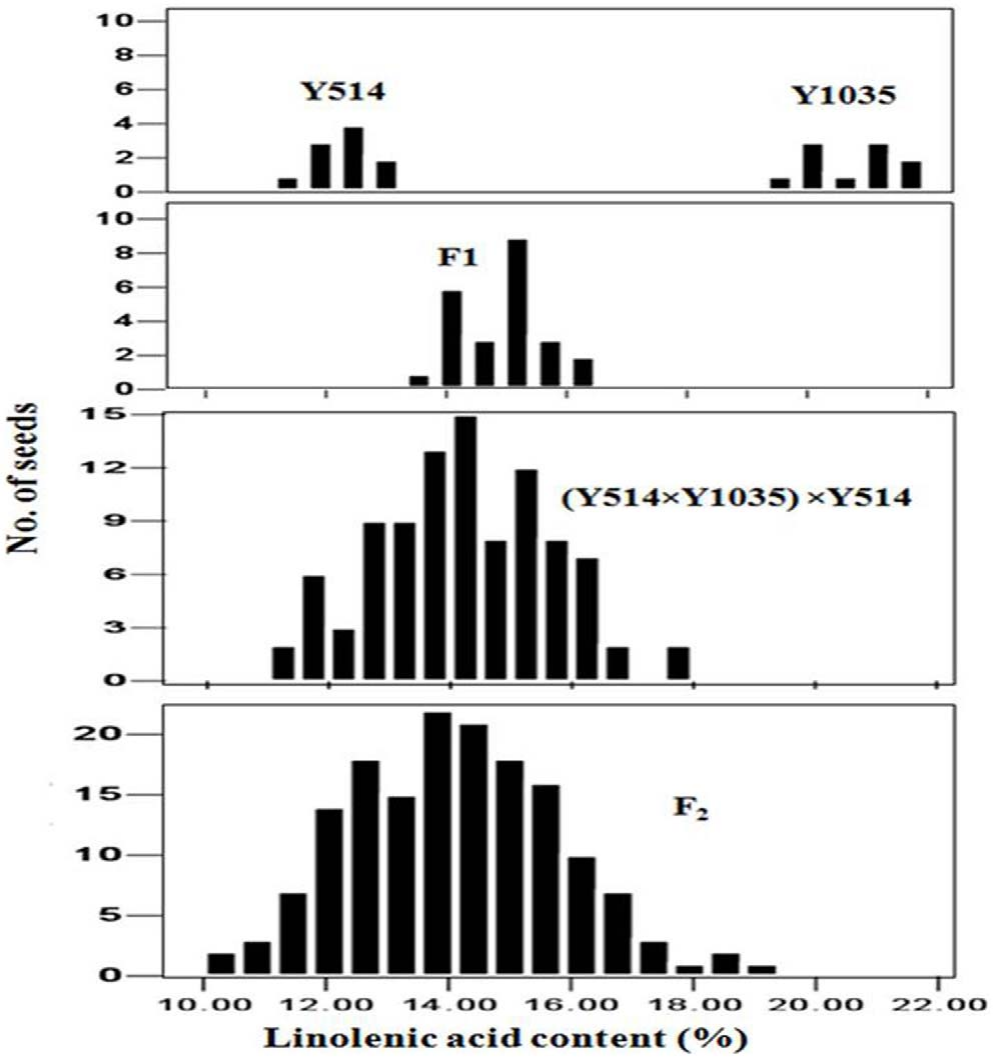
Frequency distributions of linolenic acid contents in individual seeds of Y514, Y11035, F_1_, (Y514×Y1035)×Y514 and F_2_ populations.

The C18∶3 content of the F_1_ seeds was significantly higher than the mid-parent value in the crosses of Y1127 (low)×Y1035 (high) (t = 3.84, *p*<0.01) and Y1127 (low)×Y514 (medium) (t = 5.62, *p*<0.01) (Table 1, Figure 1 and 2), suggesting a partial dominance of the high/medium over low C18∶3 content. However, in the cross of Y514 (medium)×Y1035 (high) the F_1_ seeds had significantly lower C18∶3 content (15.3%) than the mid-parent value of 16.5% (t = 6.98, *p*<0.01) (Table 1, Figure 3), indicating a partial dominance of the medium over high C18∶3 content.

The BC_1_ seeds of (Y1127×Y1035)×Y1127 were classified into two groups: seeds with medium to high (5.4–19.1%) C18∶3 content and seeds with low (3.0–4.9%) C18∶3 content (Figure 1), fitting with a segregation ratio of 3∶1 (χ^2^ = 2.00, *p* = 0.16). The F_2_ seeds of Y1127×Y1035 ranged from 2.9% to 20.4% in C18∶3 content (Figure 1) with a segregation ratio of 15∶1 (seeds with 4.5–20.4% versus seeds with 2.9–4.3% C18∶3 content) (χ^2^ = 3.07, *p* = 0.08). Therefore, the segregation patterns of C18∶3 content in the BC_1_ and F_2_ populations supported a digenic inheritance model in this cross.

The BC_1_ seeds of (Y1127×Y514)×Y1127 showed a segregation ratio of 1∶1 (seeds with 2.7–5.2% versus seeds with 6.4–9.7% C18∶3 content) (Figure 2) (χ^2^ = 3.38, *p* = 0.07), suggesting that the C18∶3 content was controlled by one gene locus in this cross. The F_2_ seeds of Y1127×Y514 showed a continuous distribution ranging from 3.0% to 16.5% in the C18∶3 content (Figure 2). The BC_1_ seeds of (Y514×Y1035)×Y514 and the F_2_ seeds of Y514×Y1035 exhibited a continuous frequency distribution in the C18∶3 content (Figure 3). Therefore, it was not possible to classify the seeds into discrete groups.

### Two QTLs Accounting for the Variation of C18∶3 Content are Mapped to Linkage Groups Sal02 and Sal10, Respectively

**Figure 4:**
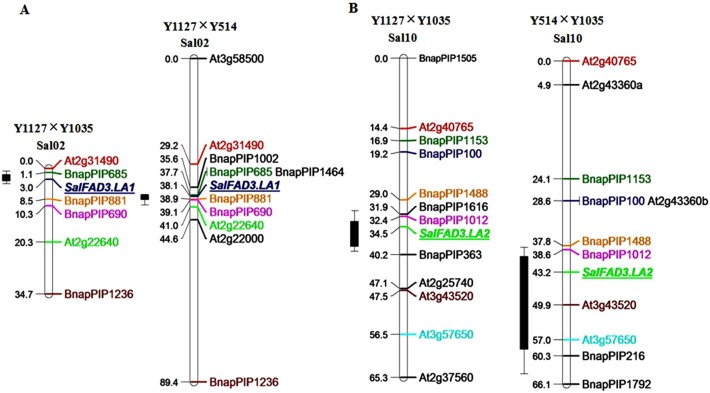
Mapping QTLs controlling C18∶3 content. A. The QTL in Sal02 was located between BnapPIP685 and BnapPIP881 in Y1127×Y1035 and Y1127×Y514. B. The QTL in Sal10 was located between BnapPIP1012 and BnapPIP363 in Y1127×Y1035, and between BnapPIP1012 and At3g43520 in Y514×Y1035. 1-LOD and 2-LOD supporting intervals of each C18∶3 QTL were marked by thick and thin bars, respectively. The *SalFAD3.LA1* and *SalFAD3.LA2* genes co-localized with their C18∶3 QTL peaks in the linkage groups Sal02 and Sal10.

In the F_2_ population of Y1127 (low)×Y1035 (high), eighteen ILP primer pairs were polymorphic between the high (16.6–20.4%) and low (2.9–4.0%) C18∶3 bulks and generated 18 markers (Table 2). The 18 markers were mapped to two linkage groups, each of which carried one QTL for the C18∶3 content (Figure 4). Based on the common ILP markers, the two linkage groups were revealed to be Sal02 and Sal10 of the constructed *S. alba* map [28]. One QTL (LOD = 45.43) accounting for 73.0% of the total variation of C18∶3 content was localized between BnapPIP685 and BnapPIP881 in Sal02 (Figure 4). The other QTL (LOD = 9.28) responsible for 23.4% of the total variation was located between BnapPIP1012 and BnapPIP363 in Sal10 (Figure 4). Together, the two QTLs explained 96.4% of the total variation for C18∶3 content in the F_2_ population.

**Table 2:** Polymorphic ILP primers used for regional linkage mapping of C18∶3 content in the three F_2_ populations of Y1127×Y1035, Y1127×Y514 and Y514×Y1035.

Primer Name	Locus Name	Y1127×Y1035		Y1127×Y514		Y514×Y1035	
		marker type	χ2 Value*	Marker type	χ2 Value	Marker type	χ2 Value
At2g22640	At2g22640	Codominant	χ^2^ = 2.15	Codominant	χ^2^ = 0.03		-
At2g31490	At2g31490	Codominant	χ^2^ = 0.00	Codominant	χ^2^ = 0.21		-
BnapPIP1236	BnapPIP1236	Dominant	χ^2^ = 0.21	Dominant	χ^2^ = 0.61		-
BnapPIP685	BnapPIP685	Dominant	χ^2^ = 1.57	Dominant	χ^2^ = 0.94		-
BnapPIP690	BnapPIP690	Dominant	χ^2^ = 1.28	Dominant	χ^2^ = 0.63		-
BnapPIP881	BnapPIP881	Codominant	χ^2^ = 2.74	Codominant	χ^2^ = 0.41		-
At2g40765	At2g40765	Codominant	χ^2^ = 1.30	-	-	Dominant	χ^2^ = 1.90
At3g43520	At3g43520	Codominant	χ^2^ = 0.46	-	-	Dominant	χ^2^ = 0.83
At3g57650	At3g57650	Codominant	χ^2^ = 0.20	-	-	Codominant	χ^2^ = 3.27
BnapPIP100	BnapPIP100	Dominant	χ^2^ = 0.10	-	-	Dominant	χ^2^ = 1.02
BnapPIP1012	BnapPIP1012	Dominant	χ^2^ = 0.30	-	-	Dominant	χ^2^ = 2.70
BnapPIP1153	BnapPIP1153	Codominant	χ^2^ = 1.28	-	-	Codominant	χ^2^ = 1.16
BnapPIP1488	BnapPIP1488	Dominant	χ^2^ = 0.05	-	-	Dominant	χ^2^ = 1.63
At2g25740	At2g25740	Codominant	χ^2^ = 0.67	-	-	-	-
At2g37560	At2g37560	Codominant	χ^2^ = 1.43	-	-	-	-
BnapPIP1505	BnapPIP1505	Codominant	χ^2^ = 3.27	-	-	-	-
BnapPIP1616	BnapPIP1616	Codominant	χ^2^ = 0.68	-	-	-	-
BnapPIP363	BnapPIP363	Dominant	χ^2^ = 1.54	-	-	-	-
At2g22000	At2g22000	-	-	Codominant	χ^2^ = 1.13	-	-
At3g58500	At3g58500	-	-	Codominant	χ^2^ = 1.70	-	-
BnapPIP1002	BnapPIP1002	-	-	Codominant	χ^2^ = 1.08	-	-
BnapPIP1464	BnapPIP1464	-	-	Dominant	χ^2^ = 1.02	-	-
At2g43360	At2g43360a	-	-	-	-	Dominant	χ^2^ = 0.68
	At2g43361b	-	-	-	-	Codominant	χ^2^ = 0.33
BnapPIP1792	BnapPIP1792	-	-	-	-	Codominant	χ^2^ = 0.53
BnapPIP216	BnapPIP216	-	-	-	-	Dominant	χ^2^ = 0.47

*: Codominant markers: Expected Mendelian segregation of 1∶2∶1, χ2 (0.05, 2) = 5.99; Dominant marker: Expected Mendelian segregation of 3∶1, χ2 (0.05, 1) = 3.84.

In the F_2_ population of Y1127 (low)×Y514 (medium), 10 polymorphic ILP primer pairs between the low (3.0–4.0%) and medium (14.5–16.5%) C18∶3 bulks produced 10 markers (Table 2). The 10 markers were all mapped to one linkage group corresponding to Sal02. The QTL (LOD = 46.53) was localized between BnapPIP685 and BnapPIP881 in the linkage group (Figure 4). In the F_2_ population of Y514 (medium)×Y1035 (high), 11 markers were generated by 10 polymorphic primer pairs between the medium (10.4–11.6%) and high (16.7–19.2%) C18∶3 bulks. The 11 markers were mapped to the linkage group Sal10. The QTL (LOD = 6.09) was located between BnapPIP1012 and At3g43520 in Sal10 (Figure 4). The two *FAD3* gene loci controlling the QTLs in Sal02 and Sal10 were referred to as *SalFAD3.LA1* and *SalFAD3.LA2*, respectively. The dominant and recessive alleles of the *SalFAD3.LA1* gene were accordingly designated as *LA^1^* and *la^1^*, while that of the *SalFAD3.LA2* gene as *LA^2^* and *la^2^*. Therefore, it could be inferred that the C18∶3 genotypes of Y1127 (low), Y514 (medium) and Y1035 (high) were *la^1^la^1^la^2^la^2^*, *LA^1^LA^1^la^2^la^2^* and *LA^1^LA^1^LA^2^LA^2^*, respectively.

### The *SalFAD3.LA1* and *SalFAD3.LA2* Genes are Cloned and Exhibit Differences in the Exon and Intron

**Figure 5:**
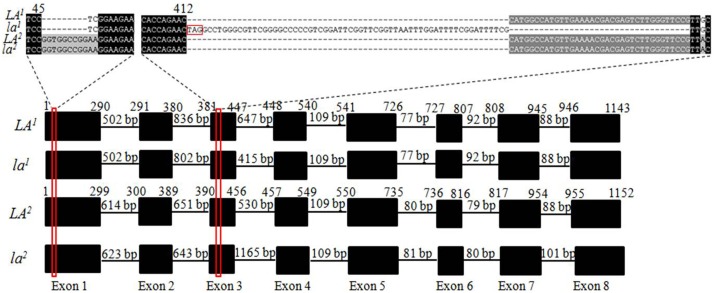
Structure of the *SalFAD3.LA1* and *SalFAD3.LA2* alleles *LA^1^*, *la^1^*, *LA^2^* and *la^2^* in yellow mustard. The black boxes represented the exons and the lines between the black boxes indicated the introns. The numbers on the top of black box of *LA^1^* (*LA^2^*) indicated the beginning and ending of each exon of *LA^1^* and *la^1^* (*LA^2^* and *la^2^*). The number above each black line indicated the intron length. The nucleotide sequences of 9 bp deletion at position 45 of exon 1 of the alleles *LA^1^* and *la^1^*, and the 64 bp insertion and the 36 bp deletion at position 412 of exon 3 of *la^1^* were displayed on the top. Allele specific markers were developed based on the variation in intron 3 of the *SalFAD3* alleles *LA^1^*, *la^1^*, *LA^2^* and *la^2^*.

The coding regions of the dominant alleles *LA^1^* and *LA^2^* were cloned from Y1035, while those of the recessive alleles, *la^1^* and *la^2^* from Y1127 using primer pair No 1 (Table S1). *LA^1^* had a coding DNA sequence (CDS) of 1143 bp encoding a polypeptide of 380 amino acids. *la^1^* had a CDS of 1171 bp. Sequence alignment with *LA^1^* indicated that *la^1^* harboured an indel involving a 64 bp insertion and a 36 bp deletion at position 412 (Figure 5 and Figure S1). A stop codon at the beginning of the 64 bp insertion might have resulted in the termination of protein translation after the 137^th^ amino acid residue. Therefore, *la^1^* is a loss-of-function allele. The 5′ flanking sequences from the translation start site were cloned for *LA^1^*and *la^1^* using the primer pair No 2 (Table S1). The 5′ fragment of *LA^1^* was 1250 bp, while that of *la^1^* was 621 bp. A 435 bp 3′ flanking sequence from the translation stop codon was cloned for *LA^1^*and *la^1^* using the primer pair No 4 (Table S1). The two alleles didn’t exhibit any differences in the cloned 3′ flanking sequences. The genomic DNA sequences of the *LA^1^* and *la^1^* were amplified using the primer pair No 5 (Table S1) which was designed based on the 5′ flanking sequence and the conserved 3′ flanking sequence specific to the candidate *SalFAD3.LA1* gene. Comparison of the coding and genomic DNA sequences indicated that the candidate *SalFAD3.LA1* gene contained 8 exons and 7 introns (Figure 5). Alignment of the genomic DNA sequences of *LA^1^* and *la^1^* revealed that *la^1^* had an insertion of 584 bp in the third exon. This insertion contained a new intron splicing site GT (Figure S4), which resulted in a 64 bp insertion and a 36 bp deletion (nucleotide 412–447) at position 412 in the CDS (Figure 5). The inserted fragment contained a 5 bp direct repeat (5′-AGAAC-3′) at each end, which is a typical LTR retroelement insertion site (Figure S4). In addition to differences in the CDS, *LA^1^* and *la^1^* exhibited variation in the length of the introns (Figure 5).

Both *LA^2^* and *la^2^* had a CDS of 1152 bp encoding a polypeptide of 383 amino acids (Figure S1 and S5). Six point mutations at positions 567, 579, 666, 699, 777 and 1059 were observed in the CDS of *la^2^* when compared with that of *LA^2^*, but did not lead to any amino acid changes. The 5′ flanking sequences from the translation start site were cloned for *LA^2^* and *la^2^* using the primer pair No 3 (Table S1). The 5′ flanking fragments of the two alleles were 444 nucleotides in length and were similar in sequence. A 435 bp 3′ flanking sequence from the translation stop codon was cloned for *LA^2^* and *la^2^* using the primer pair No 4 (Table S1). The two alleles didn’t show any differences in the cloned 3′ flanking sequences. The genomic DNA sequences of *LA^2^* and *la^2^* were cloned using primer pair No 6 (Table S1) which was designed based on the 5′ flanking sequence and the conserved 3′ flanking sequence specific to the candidate *SalFAD3.LA2* gene (Figure S3). Comparison of the coding and genomic DNA sequences indicated that the candidate *SalFAD3.LA2* gene also contained 8 exons and 7 introns (Figure 5). Variation in the length of the introns was observed between *LA^2^* and *la^2^* (Figure 5). For instance, the third intron of *LA^2^* was 530 bp, while that of *la^2^* was 1165 bp.

Sequence alignment of *LA^1^* and *LA^2^* indicated that *LA^1^* harboured a 9 bp deletion at position 46 (Figure 5 and Figure S1), which resulted in the loss of the three amino acids glycine-arginine-lysine at position 16. In addition, 77 point mutations were observed between *LA^1^* and *LA^2^* (Figure S1), of which 19 mutations led to amino acid changes (Figure S5). The candidate *SalFAD3.LA1* and *SalFAD3.LA2* genes exhibited differences in the cloned 5′ flanking sequences (Figure S2), but had the same 3′ flanking sequences. Variation in the length of the introns was observed among the four alleles *LA^1^*, *la^1^*, *LA^2^* and *la^2^* (Figure 5).

**Figure 6:**
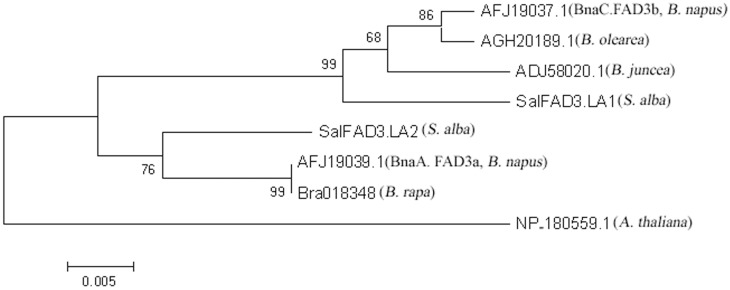
Phylogentic relationship based on the polypeptide sequences among the *LA^1^* allele of the *SalFAD3.LA1* gene, the *LA^2^* allele of the *SalFAD3.LA2* gene, and the *FAD3* genes of *Brassica* species and *Arabidopsis*.

Phylogenetic analysis based on the polypeptide sequences encoded by *LA^1^* and *LA^2^* implied that *SalFAD3.LA1* and *SalFAD3. LA2* genes in yellow mustard were clustered with *FAD3* genes in *Brassica* species (Figure 6). The *SalFAD3.LA1* gene was grouped together with the *FAD3* genes of *B. oleracea* (Genbank accession No.AGH20189), the C genome in *B. napus* (*BnaC.FAD3b*, Genbank accession No.AFJ19037.1) and *B. juncea* (Genbank accession No.ADJ58020.1), whereas the *SalFAD3. LA2* gene was in the same cluster with the *FAD3* genes of *B. rapa* (*BraA.FAD3a*, BRAD accession No. Bra018348) and the A genome in *B. napus* (*BnaA.FAD3a*, Genbank accession No.AFJ19039.1).

### Co-segregation of the *SalFAD3.LA1* and *SalFAD3.LA2* Allele-specific Markers with C18∶3 Contents in the F_2_ Populations

**Figure 7:**
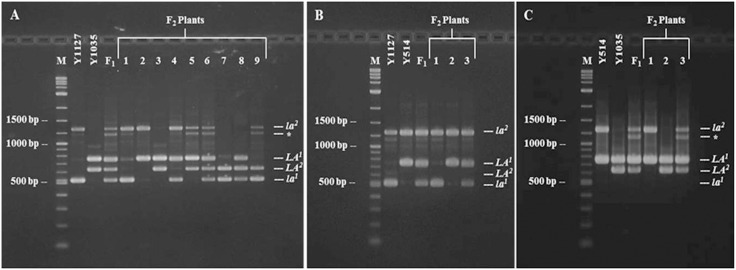
Identification of different C18∶3 genotypes based on the *SalFAD3.LA1* and *SalFAD3.LA2* allele-specific markers in the F_2_ populations of the three crosses Y1127×Y1035 (A), Y1127×Y514 (B) and Y514×Y1035 (C). M: DNA ladder. Y1127: Low C18∶3 line (*la^1^la^1^la^2^la^2^*). Y1035: high C18∶3 line (*LA^1^LA^1^LA^2^LA^2^*). A. F_1_ (Y1127×Y1035): *LA^1^la^1^LA^2^la^2^*. Lane 1: *la^1^la^1^la^2^la^2^*; Lane 2: *LA^1^LA^1^la^2^la^2^*; Lane 3: *LA^1^LA^1^LA^2^LA^2^*; Lane 4: *LA^1^la^1^la^2^la^2^*; Lane 5: *LA^1^LA^1^LA^2^la^2^*; Lane 6: *LA^1^la^1^LA^2^la^2^*; Lane 7: *la^1^la^1^LA^2^LA^2^*; Lane 8: *LA^1^la^1^LA^2^LA^2^*; Lane 9: *la^1^la^1^LA^2^la^2^*. B. F_1_ (Y1127×Y1035): *LA^1^la^1^la^2^la^2^*; Lane 1: *la^1^la^1^la^2^la^2^*; Lane 2: *LA^1^LA^1^la^2^la^2^*; Lane 3: *LA^1^la^1^la^2^la^2^*. C. F_1_ (Y514×Y1035): *LA^1^LA^1^LA^2^la^2^*; Lane 1: *LA^1^LA^1^la^2^la^2^*; Lane 2: *LA^1^LA^1^LA^2^LA^2^*; Lane 3: *LA^1^LA^1^LA^2^la^2^*. * indicated new band. The different C18∶3 genotypes were identified using primer No 12 (SalFAD3 F and SalFAD3 R) (Table S1) designed based on the variation in intron 3 of the *SalFAD3* alleles *LA^1^*, *la^1^*, *LA^2^* and *la^2^*.

Primer pair 12 (Table S1) produced co-dominant markers of 742 bp, 510 bp, 626 bp and 1273 bp specific for *LA^1^*, *la^1^*, *LA^2^* and *la^2^*, respectively, which co-segregated with the C18∶3 content in all of the F_2_ populations. In the cross of Y1127 (low)×Y1035 (high), all of the nine possible genotypes were identified using the markers specific for *LA^1^*, *la^1^*, *LA^2^* and *la^2^* (Figure 7A; Table 3). The homozygous F_2_ plants (*LA^1^LA^1^LA^2^LA^2^*) had a significantly higher C18∶3 content (average: 17.1%) than the heterozygous F_2_ plants of *LA^1^la^1^LA^2^LA^2^* (average: 13.1%) (t = 6.12, *p*<0.01) and of *LA^1^LA^1^LA^2^la^2^* (average: 15.5%) (t = 2.23, *p* = 0.04) (Table 3). The homozygous F_2_ plants of *LA^1^LA^1^la^2^la^2^* had a higher average C18∶3 content (14.0%) than those of *la^1^la^1^LA^2^LA^2^* (average: 9.1%) (t = 5.81, *p*<0.01). In the cross of Y1127 (low)×Y514 (medium), the three genotypes for C18∶3 content were differentiated with the markers specific for *LA^1^* and *la^1^* (Figure 7B; Table 3). The average C18∶3 content of the homozygous F_2_ plants (*LA^1^LA^1^la^2^la^2^*) was 12.7%, which was significantly higher than the heterozygous F_2_ plants (*LA^1^la^1^la^2^la^2^*, average: 9.2%) (t = 5.02, *p*<0.01) (Table 3). In the cross of Y514 (medium)×Y1035 (high), the markers specific for *LA^2^* and *la^2^* distinguished the three C18∶3 genotypes (Figure 7C; Table 3). The homozygous F_2_ plants (*LA^1^LA^1^LA^2^LA^2^*) had an average C18∶3 content of 15.8%, which was higher than the heterozygous F_2_ plants (*LA^1^LA^1^LA^2^la^2^*, average: 13.8%) (t = 2.23, *p* = 0.04) (Table 3). The *SalFAD3.LA1* and *SalFAD3.LA2* genes co-localized with the QTL peaks on Sal02 and Sal10, respectively (Figure 4). A new band was observed in the F_1_ and F_2_ plants with the heterozygote genotype of *LA^2^la^2^* (Figs. 7A and 7C).

**Table 3:** Co-segregation of the *SalFAD3.LA1* and *SalFAD3.LA2* allele-specific markers with C18∶3 contents in the F_2_ populations of Y1127×Y1035, Y1127×Y514 and Y514×Y1035.

F_2_ populations	Allele-specific Markers				Genotype	No. of Plants	C18∶3 Content (% of total fatty acids)	
	*LA^1^*	*la^1^*	*LA^2^*	*la^2^*			Mean	Range
Y1127×Y1035	+	−	+	−	*LA^1^LA^1^LA^2^LA^2^*	9	17.1	15.4–20.4
	+	−	+	+	*LA^1^LA^1^LA^2^la^2^*	22	15.5	11.1–18.7
	+	−	−	+	*LA^1^LA^1^ la^2^la^2^*	5	14.0	10.0–16.0
	+	+	+	−	*LA^1^la^1^LA^2^LA^2^*	14	13.1	10.4–15.5
	+	+	+	+	*LA^1^la^1^LA^2^la^2^*	44	11.1	8.5–13.4
	+	+	−	+	*LA^1^la^1^la^2^la^2^*	20	10.2	8.3–12.5
	−	+	+	−	*la^1^la^1^LA^2^LA^2^*	12	9.1	7.7–10.6
	−	+	+	+	*la^1^la^1^LA^2^la^2^*	19	5.7	3.1–7.5
	−	+	−	+	*la^1^la^1^la^2^la^2^*	12	3.9	2.9–4.5
Y1127×Y514	+	−	−	+	*LA^1^LA^1^ la^2^la^2^*	36	12.7	8.6–16.5
	+	+	−	+	*LA^1^la^1^la^2^la^2^*	82	9.2	5.6–14.8
	−	+	−	+	*la^1^la^1^la^2^la^2^*	37	4.6	3.0–5.9
Y514×Y1035	+	−	+	−	*LA^1^LA^1^LA^2^LA^2^*	37	15.8	13.7–19.2
	+	−	+	+	*LA^1^LA^1^LA^2^la^2^*	90	13.8	10.1–16.0
	+	−	−	+	*LA^1^LA^1^ la^2^la^2^*	30	12.9	10.4–15.0

## Discussion

The present paper reported on the inheritance and QTL mapping of C18∶3 content as well as molecular characterization of the *FAD3* genes in yellow mustard. Linolenic acid content was controlled by the nuclear genotype of the embryo in yellow mustard as reported in *B. napus* [8]. Two nuclear gene loci were detected and functioned independently and additively to determine the total C18∶3 content in the seeds. However, maternal effects on the C18∶3 content couldn’t be ruled out since appropriate progeny tests were not performed in the present study. QTL analysis further revealed that the two gene loci *SalFAD3.LA1* and *SalFAD3.LA2* had a different magnitude of effect and together explained 96.4% of the total variation for C18∶3 content. The residual 3.6% variation of C18∶3 content beyond the two QTLs could be resulted from maternal and environmental effects. It has been reported that temperature, maternal genotype and cytoplasm have effects on C18∶3 content in *B. napus* [8]–[10]. The duplication of the *FAD3* gene provides additional evidence that yellow mustard is a secondary polyploid species as revealed by molecular studies [29], [30]. The two linkage groups Sal02 and Sall0 containing the *SalFAD3.LA1* and *SalFAD3.LA2* genes, did not share any common ILP markers, suggesting the occurrence of extensive genomic changes during the speciation of yellow mustard.

Molecular cloning and sequencing indicated that the *SalFAD3.LA1* and *SalFAD3.LA2* genes contained 8 exons and 7 introns in yellow mustard, which is in agreement with that in *B. napus* [12] and *A. thaliana* (Locus: AT2G29980, TAIR) [31]. However, the molecular mechanism underlying the naturally occurring C18∶3 variant in yellow mustard was different from that of the EMS-induced C18∶3 variant in *B. napus* and *B. oleracea*. The *FAD3* gene with reduced C18∶3 content resulted from SNP mutations in *B. napus* [11], [12] and *B. oleracea* [32]. However, the recessive allele *la^1^* of the *SalFAD3.LA1* gene was a loss-of-function mutant due to an insertion of 584 bp in exon 3. The inserted fragment contained a typical LTR retroelement insertion site (5′-AGAAC-3′) at each end, suggesting that the inserted fragment might be a remnant of a transposable element which had undergone a deletion following the insertion event. The recessive allele *la^2^* of the *SalFAD3.LA2* gene was functional and had a CDS encoding the same polypeptide sequence when compared with the dominant allele *LA^2^*. However, *la^2^* was different in intron sequence. It remains to be investigated why *LA^2^* and *la^2^* controlled a different C18∶3 content. The *SalFAD3.LA1* and *SalFAD3.LA2* allele-specific markers proved to be useful for identification of different C18∶3 genotypes in the present study.

The phylogenetic analysis based on the polypeptide sequences indicated that the *LA^1^* and *LA^2^* genes in yellow mustard were clustered with the *FAD3* genes in *Brassica* species and *A. thaliana*. Interestingly, *LA^1^* and *LA^2^* were clustered into different groups. *LA^1^* was grouped together with the *FAD3* genes of *B. oleracea* and the C genome in *B. napus,* whereas *LA^2^* was in the same cluster with the *FAD3* gene of *B. rapa* and the A genome in *B. napus*. In our study, the *LA^1^* gene controlled a higher C18∶3 content than the *LA^2^* gene. It was reported that the *FAD3* gene of the C genome in *B. napus* also contributed more to the total C18∶3 content than that of the A genome [11], [12]. This suggested that the molecular divergence of the *LA^1^* and *LA^2^* genes occurred before the speciation of yellow mustard and *Brassica* species.

In conclusion, our study revealed the existence of two *FAD3* gene loci contributing to the genetic variation of linolenic acid content in yellow mustard. The *SalFAD3.LA1* gene was located in the linkage group Sal02, while the *SalFAD3.LA2* gene in Sal10. We have cloned the *SalFAD3.LA1* and *SalFAD3.LA2* genes and developed allele-specific markers for the detection of desirable genotypes, which will be valuable for marker assisted breeding in yellow mustard.

## Supporting Information

Figure S1 **Alignment of the coding DNA sequences of the** ***SalFAD3.LA1*** **alleles** ***LA^1^*** **,** ***la^1^*** **and the** ***SalFAD3.LA2*** **alleles** ***LA^2^*** **and** ***la^2^*** **in yellow mustard.** The nucleotide sequence alignment was carried out using ClustalW2 (http://www.ebi.ac.uk/Tools/msa/clustalW2/).
(PDF)

Figure S2 **Alignment of the 5′ upstream sequences of the** ***SalFAD3.LA1*** **and** ***SalFAD3.LA2*** **genes in yellow mustard.** *UP-LA^1^* and *UP-la^1^* represented the 5′ upstream sequences of the alleles *LA^1^* and *la^1^* of the *SalFAD3.LA1* gene. *UP-LA^2^* and *UP-la^2^* indicated the 5′ upstream sequences of the alleles *LA^2^* and *la^2^* of the *SalFAD3.LA2* gene. The nucleotide sequence alignment was carried out using ClustalW2 (http://www.ebi.ac.uk/Tools/msa/clustalw2/).
(PDF)

Figure S3 **PCR amplification of the** ***SalFAD3.LA1*** **and** ***SalFAD3.LA2*** **genes.** A. PCR amplification of the genomic DNA sequences of *SalFAD3.LA1* gene using the primer pair No 5 (Table S1). Lanes 1–2: 4268 bp fragment of *la^1^* from Y1127; Lanes 3–6: 4534 bp fragment of *LA^1^* from Y1035. B. PCR amplification of the genomic DNA sequences of *SalFAD3.LA2* gene using the primer pair No 6 (Table S1). Lanes 1–4: 4688 bp fragment of *la^2^* from Y1127; Lanes 5–6: 4042 bp fragment of *LA^2^* from Y1035.
(PDF)

Figure S4 **Nucleotide sequences of intron 3 and its flanking of** ***SalFAD3.LA1*** **and** ***SalFAD3.LA2*** **genes in yellow mustard.** The sequence of exon 3 was underlined in red while that of exon 4 was lined in blue. The nucleotide sequence of the inserted fragment in exon 3 of *la^1^* was underlined in pink. The new intron splicing site GT in the inserted fragment was indicated in green rectangle box. The nucleotides in blue rectangle box indicated the inserted fragment that remained in the CDS of exon 3 of *la^1^*. The nucleotides in red rectangle box indicated the 5 bp direct repeat (5′-AGAAC-3′). The first and the last nucleotides of intron 3 were indicated by arrowhead and arrow, respectively. The intron 3 of *LA^1^*, *la^1^ LA^2^*, *la^2^* are 647 bp, 415 bp, 530 bp and 1165 bp in length, respectively.
(PDF)

Figure S5 **Amino acid sequences encoded by the** ***SalFAD3.LA1*** **allele** ***LA^1^*** **and the** ***SalFAD3.LA2*** **allele** ***LA^2^*** **of yellow mustard, AFJ19039.1 (** ***BraA.FAD3a*** **) and AFJ19037.1 (** ***BnaC.FAD3b*** **) of** ***B. napus*** **, AGH20189.1 of** ***B. oleracea*** **, ADJ58020.1 of** ***B. juncea*** **, Bra018348 of** ***B. rapa*** **, NP_180559.1 of** ***A. thaliana*** **.** The amino acid sequence alignment was carried out by ClustalW2 (http://www.ebi.ac.uk/Tools/msa/clustalw2/).
(PDF)

Table S1 **Primers used in this study.**
(PDF)
