# Supplementary material for: Detection and Molecular Characterization of Two FAD3 Genes Controlling Linolenic Acid Content and Development of Allele-Specific Markers in Yellow Mustard (Sinapis alba)
Source: PLoS One. 2014 May 13;9(5):e97430. doi: 10.1371/journal.pone.0097430 (PMC4019595; doi:10.1371/journal.pone.0097430)
Supplement: Figure S1 — Alignment of the coding DNA sequences of the SalFAD3.LA1 alleles LA1 , la1 and the SalFAD3.LA2 alleles LA2 and la2 in yellow mustard. The nucleotide sequence alignment was carried out using ClustalW2 (http://www.ebi.ac.uk/Tools/msa/clustalW2/). (PDF) [file pone.0097430.s001.pdf]

```
                *         20         *         40         *         60         *         80
LA1 : ATGGTTGTCGCTATGGACCAGCGTAGCAATGTGAACGGAGATTCC---------TCGGAAGAAAGGTTTGATCCGAGCGCACAA :  75
la1 : ATGGTTGTTGCTATGGACCAGCGTAGCAATGTGAACGGAGATTCC---------TCGGAAGAAAGGTTTGATCCGAGCGCACAA :  75
LA2 : ATGGTTGTTGCTATGGACCAGCGCAGCAATGTGAACGGAGTCTCCGGTGGCCGGAAGGAAGAAGGGTTTGATCCAAGCGCACAG :  84
la2 : ATGGTTGTTGCTATGGACCAGCGCAGCAATGTGAACGGAGTCTCCGGTGGCCGGAAGGAAGAAGGGTTTGATCCAAGCGCACAG :  84

          *        100         *        120         *        140         *        160
LA1 : CCACCGTTTAAGATCGGAGACATAAGGGCGGCGATTCCTAAGCATTGTTGGGTAAAAAGTCCTTTGAGATCCATGAGCTACGTC : 159
la1 : CCACCGTTTAAGATCGGAGATATAAGGGCGGCGATTCCTAAGCATTGTTGGGTTAAGAGTCCTTTGAGATCCATGAGCTACGTC : 159
LA2 : CCACCGTTTAAGATCGGAGATATAAGAGCGGCGATTCCTAAGCATTGTTGGGTGAAGAGTCCTTTGAGATCTATGAGCTACGTC : 168
la2 : CCACCGTTTAAGATCGGAGATATAAGAGCGGCGATTCCTAAGCATTGTTGGGTGAAGAGTCCTTTGAGATCTATGAGCTACGTC : 168

        *        180         *        200         *        220         *        240         *
LA1 : GCGAGAGACATTTTCGCCGTCGTGGCTCTCGCCGTCGCCGCCGTGTATTTTAATAGCTGGCTCTTCTGGCCTCTTTATTGGGCC : 243
la1 : GCCAGAGACATTTTCGCCGTCGTGGCTCTCGCCGTCGCCGCCGTGTATTTTAATAGCTGGCTCTTCTGGCCTCTTTATTGGGCC : 243
LA2 : GTGAGAGACATTGTCGCCGTCGCGGCTATGGCCATGGCCGCCGTGTATTTTGATAGCTGGTTCCTCTGGCCACTCTACTGGGCT : 252
la2 : GTGAGAGACATTGTCGCCGTCGCGGCTATGGCCATGGCCGCCGTGTATTTTGATAGCTGGTTCCTCTGGCCACTCTACTGGGCT : 252

            260         *        280         *        300         *        320         *
LA1 : GCCCAAGGAACCCTTTTCTGGGCTATCTTCGTACTCGGCCACGACTGTGGACATGGGAGCTTCTCAGACATTCCTCTTCTGAAT : 327
la1 : GCCCAAGGAACCCTTTTCTGGGCTATCTTCGTACTCGGCCACGACTGTGGACATGGGAGCTTCTCAGACATTCCTCTTCTGAAT : 327
LA2 : GCCCAAGGAACTCTTTTCTGGGCCATCTTCGTTCTGGGCCACGACTGTGGACATGGGAGTTTCTCAGACATTCCTCTGCTGAAC : 336
la2 : GCCCAAGGAACTCTTTTCTGGGCCATCTTCGTTCTGGGCCACGACTGTGGACATGGGAGTTTCTCAGACATTCCTCTGCTGAAC : 336

        340         *        360         *        380         *        400         *        420
LA1 : ACTGTGGTTGGTCATATTCTTCATTCCTTTATCCTCGTTCCATACCATGGTTGGAGAATAAGCCATCGGACACACCACCAGAAC : 411
la1 : ACTGTGGTTGGTCATATTCTTCATTCCTTTATCCTCGTTCCATACCATGGTTGGAGAATAAGCCATCGGACACACCACCAGAAC : 411
LA2 : AGTGTGGTTGGTCACATTCTTCATTCATTCATCCTCGTTCCTTACCATGGTTGGAGAATAAGCCATCGGACACACCACCAGAAC : 420
la2 : AGTGTGGTTGGTCACATTCTTCATTCATTCATCCTCGTTCCTTACCATGGTTGGAGAATAAGCCATCGGACACACCACCAGAAC : 420

                *        440         *        460         *        480         *        500
LA1 : ----------------------------------------------------------------CATGGCCATGTTGAAAACGA : 431
la1 : TAGGCCTGGGCGTTCGGGGCCCCCGTCGGATTCGGTTCGGTTAATTTGGATTTTCGGATTTTCG-------------------- : 475
LA2 : ----------------------------------------------------------------CATGGCCATGTTGAAAACGA : 440
la2 : ----------------------------------------------------------------CATGGCCATGTTGAAAACGA : 440

          *        520         *        540         *        560         *        580
LA1 : CGAGTCTTGGGTTCCGTTGCCAGAAAAATTATACAAGAAATTGTCCCACAGTACACGGATGCTCAGATACACTGTCCCTCTCCC : 515
la1 : ----------------TTGCCAGAAAAATTATACAAGAAATTGTCCCACAGTACACGGATGCTCAGATACACTGTCCCTCTCCC : 543
LA2 : CGAGTCTTGGGTTCCGTTACCAGAAAAGTTGTACAAGAACTTGCCCCATAGTACTCGGATGCTCAGATACACCGTCCCTCTGCC : 524
la2 : CGAGTCTTGGGTTCCGTTACCAGAAAAGTTGTACAAGAACTTGCCCCATAGTACTCGGATGCTCAGATACACCGTCCCTCTGCC : 524

        *        600         *        620         *        640         *        660         *
LA1 : CATGCTCGCTTACCCTCTCTATCTGTGGTACAGAAGTCCTGGTAAAGAAGGGTCACATTATAACCCATACAGTAGTTTATTTGC : 599
la1 : CATGCTCGCTTACCCTCTCTATCTGTGGTACAGAAGTCCTGGTAAAGAAGGGTCACATTATAACCCATACAGTAGTTTATTTGC : 627
LA2 : CATGCTCGCTTACCCGATCTATCTGTGGTACAGAAGCCCTGGGAAAGAAGGGTCGCACTTTAACCCATACAGTAGTTTATTTGC : 608
la2 : CATGCTCGCTTACCCGATCTATCTGTGGTACAGAAGCCCTGGAAAAGAAGGGTCACACTTTAACCCATACAGTAGTTTATTTGC : 608

            680         *        700         *        720         *        740         *
LA1 : TCCAAGCGAGAGAAATCTTATTGCAACTTCAACTACTTGCTGGTCCATCATGTTGGCCACTCTTGTTTATCTATCATTCCTCGT : 683
la1 : TCCAAGCGAGAGAAATCTTATTGCAACTTCAACTACTTGCTGGTCCATCATGTTGGCCACTCTTGTTTATCTATCGTTCCTCGT : 711
LA2 : TCCAAGCGAGAGAAAGCTTATTGCAACTTCAACTACTTGCTGGTCCATCATGTTGGCTACTCTTGTTTATCTATCGTTCCTCGT : 692
la2 : TCCAAGCGAGAGAAAGCTTATTGCAACTTCAACTACTTGCTGGTCCATCATGTTGGCCACTCTTGTTTATCTATCGTTCCTCGT : 692

        760         *        780         *        800         *        820         *        840
LA1 : TGGTCCATTCACAGTTCTCAAAGTTTATGGTGTTCCTTACATCATCTTTGTGATGTGGTTGGACGCTGTCACGTACTTGCATCA : 767
la1 : TGGTCCATTCACAGTTCTCAAAGTTTATGGTGTTCCTTACATTATCTTTGTGATGTGGTTGGACGCTGTCACGTACTTGCATCA : 795
LA2 : TGGTCCAGTCACTGTTCTCAAAGTCTATGGTGTTCCTTACATCATCTTTGTGATGTGGTTGGACGCTGTCACGTACTTGCATCA : 776
la2 : TGGTCCGGTCACTGTTCTCAAAGTCTATGGTGTTCCTTACATCATCTTTGTGATGTGGTTGGACGCTGTCACGTACTTGCATCA : 776

                *        860         *        880         *        900         *        920
LA1 : TCATGGTCACGATGATAAGTTGCCTTGGTACAGAGGCAAGGAATGGAGTTATTTACGTGGAGGATTAACAACTGTTGATAGAGA : 851
la1 : TCATGGTCACGATGATAAGTTGCCTTGGTACAGAGGCAAGGAATGGAGTTATTTACGTGGAGGATTAACAACTGTTGATAGAGA : 879
LA2 : TCATGGTCACGATGGGAAGTTGCCTTGGTACAGAGGCAAGGAATGGAGTTATTTACGTGGAGGATTAACAACTATTGACAGAGA : 860
la2 : CCATGGTCACGATGGGAAGTTGCCTTGGTACAGAGGCAAGGAATGGAGTTATTTACGTGGAGGATTAACAACTATTGACAGAGA : 860
```

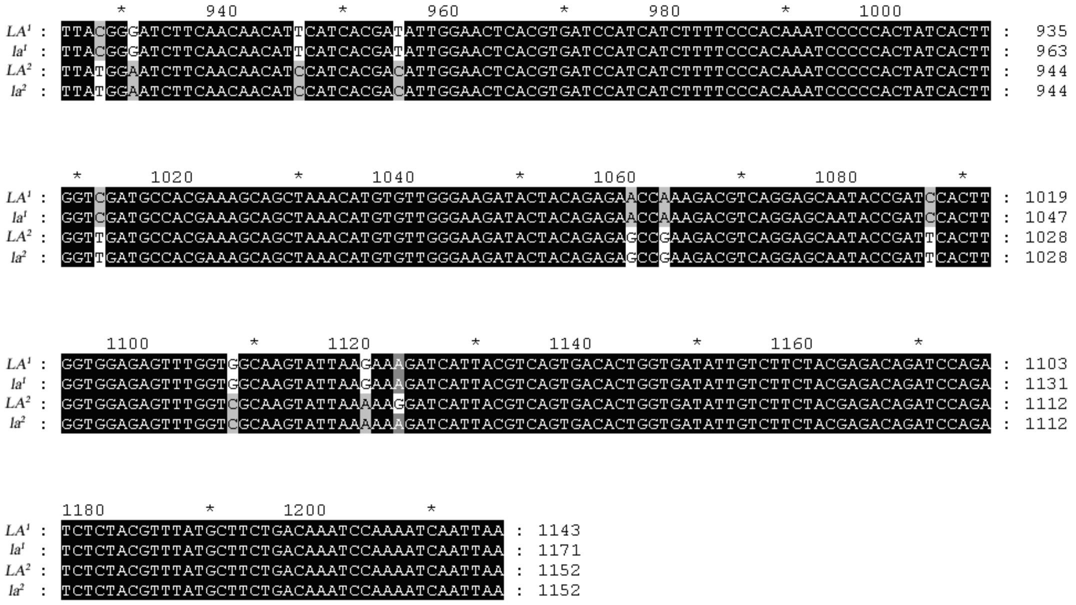

Supplementary Figure S1. Alignment of the coding DNA sequences of the *SalFAD3.LA1* alleles *LA*^{1}, *la*^{1} and the *SalFAD3.LA2* alleles *LA*^{2} and la^{2} in yellow mustard. The nucleotide sequence alignment was carried out using ClustalW2 (http://www.ebi.ac.uk/Tools/msa/clustalw2/).
