# Supplementary material for: Detection and Molecular Characterization of Two FAD3 Genes Controlling Linolenic Acid Content and Development of Allele-Specific Markers in Yellow Mustard (Sinapis alba)
Source: PLoS One. 2014 May 13;9(5):e97430. doi: 10.1371/journal.pone.0097430 (PMC4019595; doi:10.1371/journal.pone.0097430)
Supplement: Figure S2 — Alignment of the 5′ upstream sequences of the SalFAD3.LA1 and SalFAD3.LA2 genes in yellow mustard. UP-LA1 and UP-la1 represented the 5′ upstream sequences of the alleles LA1 and la1 of the SalFAD3.LA1 gene. UP-LA2 and UP-la2 indicated the 5′ upstream sequences of the alleles LA2 and la2 of the SalFAD3.LA2 gene. The nucleotide sequence alignment was carried out using ClustalW2 (http://www.ebi.ac.uk/Tools/msa/clustalw2/). (PDF) [file pone.0097430.s002.pdf]

```
                        *        20         *        40         *        60         *        80
UP-LA1 : CGACGGCCCGGGCTGGTATCGATATGAATCGTTTTTATGATTGATAAGTAGTCGAGGAAATTTTCGCAGAAACAAAATATATGG :  84
UP-la1 : CGACGGCCCGGGCTGGTATCGATATGAATCGTTTTTATGATTGATAAGTAGTCGAGGAAATTTAAGCAGAAACAAAATATATGG :  84
UP-LA2 : CGACGGCCCGGGCTGGTATC--TTCGAATC----TCAT-ATTTAT-----------GAAATT-------------AGTGTAA-- :  51
UP-la2 : CGACGGCCCGGGCTGGTATC--TTCGAATC----TCAT-ATTTAT-----------GAAATT-------------AGTGTAA-- :  51

                 *       100         *       120         *       140         *       160
UP-LA1 : AGAAGACAAACATGATATTTTCGACCACATGTATAATGTATTACTCTAAAGTAAGAAACATAAACAATTTGTATTTGGTATAAT : 168
UP-la1 : AGAAGACACACATGATATTTTCGACCACATGTATAATGCATTACTCTAAAGTAAGAAACATAAATAATTTGAATTTGGTATAAT : 168
UP-LA2 : ----------------ATTTT----------------------------------AAACGTAAACAATTTA-------TACGAC :  78
UP-la2 : ----------------ATTTT----------------------------------AAACGTAAACAATTTA-------TACGAC :  78

             *       180         *       200         *       220         *       240         *
UP-LA1 : CCTTACGTCTAAGGAAAATCTATAGAGAAAAAAGGATATAAAAATCTTGGTAAATTATAGATGCGATGGT-ACTTATACTCAAT : 251
UP-la1 : CCTTACGT-------------------------------------------AAATTATCGATGCGATGGTTACTTATACTCAAT : 209
UP-LA2 : C--------------------------------------------------AGA----------AATGGC-------------- :  88
UP-la2 : C--------------------------------------------------AGA----------AATGGC-------------- :  88

                 260         *       280         *       300         *       320         *
UP-LA1 : ACTAAGATTT-AGTTAACTAAAATCT-CTCAAAATCTTAT-----AGAAAAATTCGTAACAAATTTCTACAACTTATTTTTTCA : 328
UP-la1 : ACTAAGATTTTAGTTA--TAAAATCTTCTCAGAATCTTATTTTAAAAAAAAATTCATAACAAATTTCTACAACTTATTTTTTCA : 291
UP-LA2 : ---GAGATGT-------------TTTTCTTAGAATTTTAT-----------------AACA------------TTACTTTAGCG : 127
UP-la2 : ---GAGATGT-------------TTTTCTTAGAATTTTAT-----------------AACA------------TTACTTTAGCG : 127

             340         *       360         *       380         *       400         *       420
UP-LA1 : AAAAGAAAAATTTATA-------TAACTTAGTTTTCATCGAAATGAACGACGTAACAAAG--ATACTTTGCAC-------GCAA : 396
UP-la1 : AAAGGTAAAATTTATACTCCGTATAACTTAGTTTTCATCGAAATGAACGACGTAACAAAG--ATACTTTGCAC-------GCAA : 366
UP-LA2 : TAAAC--ACATTTCTC-------CAATTTGGTTTTCATTGCATTGAACGACGTAACAAAGTAATACACCCAACCCATTGTACAA : 202
UP-la2 : TAAAC--ACATTTCTC-------CAATTTGGTTTTCATTGCATTGAACGACGTAACAAAGTAATACACCCAACCCATTGTACAA : 202

                     *       440         *       460         *       480         *       500
UP-LA1 : AATTTACAGCAAAAATGCACATTTTATTATTCAAATAAATAAATAAAGAGCAATTTTTTAATTTTTTTTTTAACAAAAACATAC : 480
UP-la1 : AATT-ACAGCAAAAATACACATTTTATTCTTCAAATAAATAAATAAAAA----------------------------------- : 414
UP-LA2 : AAATAACAGCTAAATTACCCTTTTTATTCTTTTGATAAAGAAATAAA------------------------------------- : 249
UP-la2 : AAATAACAGCTAAATTACCCTTTTTATTCTTTTGATAAAGAAATAAA------------------------------------- : 249

                 *       520         *       540         *       560         *       580
UP-LA1 : CTCAATAAAATAAAATGACTAAAATAGTTTTTTTTTTGAAAATTTTAATTTTTAATTTTAATTTTTTTTTTTTTTAATTTTACC : 564
UP-la1 : ------------------------------------------------------------------------------------ :   -
UP-LA2 : ------------------------------------------------------------------------------------ :   -
UP-la2 : ------------------------------------------------------------------------------------ :   -

             *       600         *       620         *       640         *       660         *
UP-LA1 : CTCAAATTTTGCCACTTTAACTTTAAAGCCTAAGTCTATATCAGTTAACTCTAAAGATAAAAGCGTTTATTTACCTATTTAATA : 648
UP-la1 : ------------------------------------------------------------------------------------ :   -
UP-LA2 : ------------------------------------------------------------------------------------ :   -
UP-la2 : ------------------------------------------------------------------------------------ :   -

                 680         *       700         *       720         *       740         *
UP-LA1 : AAATATTTTAATATTTTTTAGATATTATTTTGGGCAATTTTCTTAAATAACTATTTTTAAGTTTTTGTCACAAAAATAGCTCTC : 732
UP-la1 : ------------------------------------------------------------------------------------ :   -
UP-LA2 : ------------------------------------------------------------------------------------ :   -
UP-la2 : ------------------------------------------------------------------------------------ :   -

             760         *       780         *       800         *       820         *       840
UP-LA1 : AAAAAATAAAATGACCAAAATAGATTTTTTTTTTTGAAATTTTGATTTTTTAATTTTTTTTTTTGAAATTTGAAACCCTAACCC : 816
UP-la1 : ------------------------------------------------------------------------------------ :   -
UP-LA2 : ------------------------------------------------------------------------------------ :   -
UP-la2 : ------------------------------------------------------------------------------------ :   -

                     *       860         *       880         *       900         *       920
UP-LA1 : TAAACCCACCCCTTTACTCTAAAACCTAAAATCCAAATTAGTTAACCCTAGGGATATAAATGCATATAAATGAATTTTTTAGCT : 900
UP-la1 : ------------------------------------------------------------------------------------ :   -
UP-LA2 : ------------------------------------------------------------------------------------ :   -
UP-la2 : ------------------------------------------------------------------------------------ :   -
```

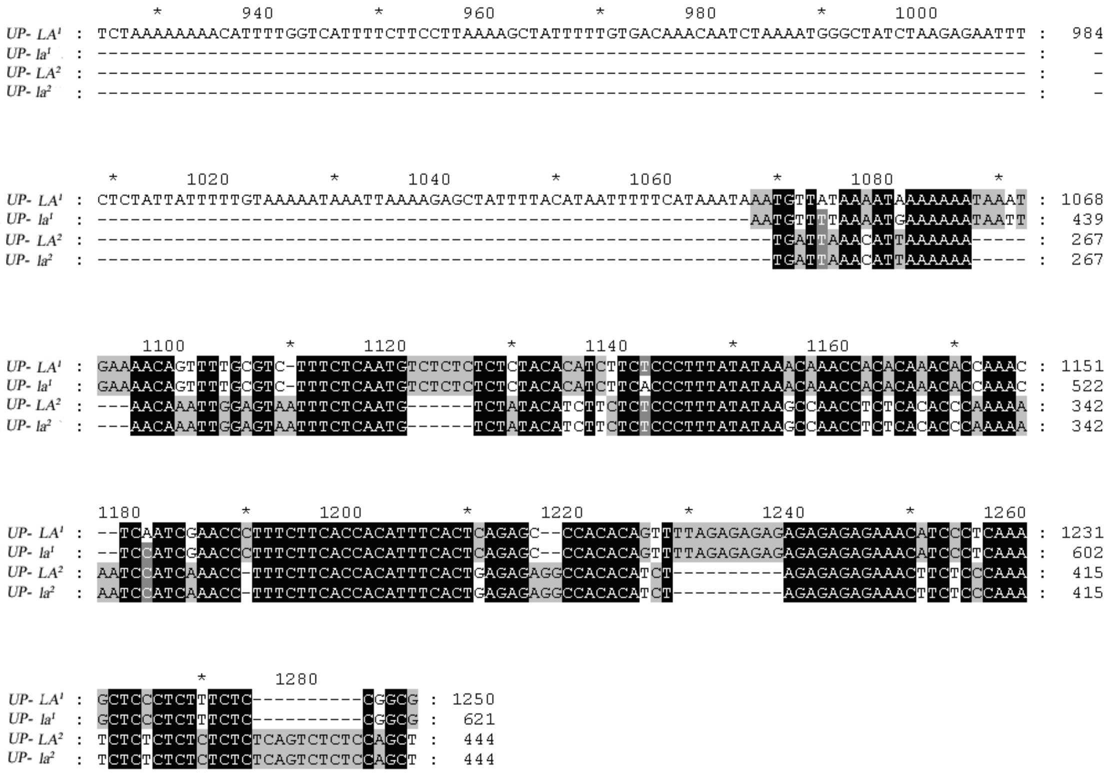

Supplementary Figure S2. Alignment of the 5' upstream sequences of the *SalFAD3.LA1* and *SalFAD3.LA2* genes in yellow mustard. *UP-LA*^{1} and *UP-la*^{1} represented the 5' upstream sequences of the alleles *LA*^{1} and *la*^{1} of the *SalFAD3.LA1* gene. *UP-LA*^{2} and *UP-la*^{2} indicated the 5' upstream sequences of the alleles *LA*^{2} and *la*^{2} of the *SalFAD3.LA2* gene. The nucleotide sequence alignment was carried out using ClustalW2 (http://www.ebi.ac.uk/Tools/msa/clustalw2/).
