# Supplementary material for: Detection and Molecular Characterization of Two FAD3 Genes Controlling Linolenic Acid Content and Development of Allele-Specific Markers in Yellow Mustard (Sinapis alba)
Source: PLoS One. 2014 May 13;9(5):e97430. doi: 10.1371/journal.pone.0097430 (PMC4019595; doi:10.1371/journal.pone.0097430)
Supplement: Figure S3 — PCR amplification of the SalFAD3.LA1 and SalFAD3.LA2 genes. A. PCR amplification of the genomic DNA sequences of SalFAD3.LA1 gene using the primer pair No 5 (Table S1). Lanes 1–2: 4268 bp fragment of la1 from Y1127; Lanes 3–6: 4534 bp fragment of LA1 from Y1035. B. PCR amplification of the genomic DNA sequences of SalFAD3.LA2 gene using the primer pair No 6 (Table S1). Lanes 1–4: 4688 bp fragment of la2 from Y1127; Lanes 5–6: 4042 bp fragment of LA2 from Y1035. (PDF) [file pone.0097430.s003.pdf]

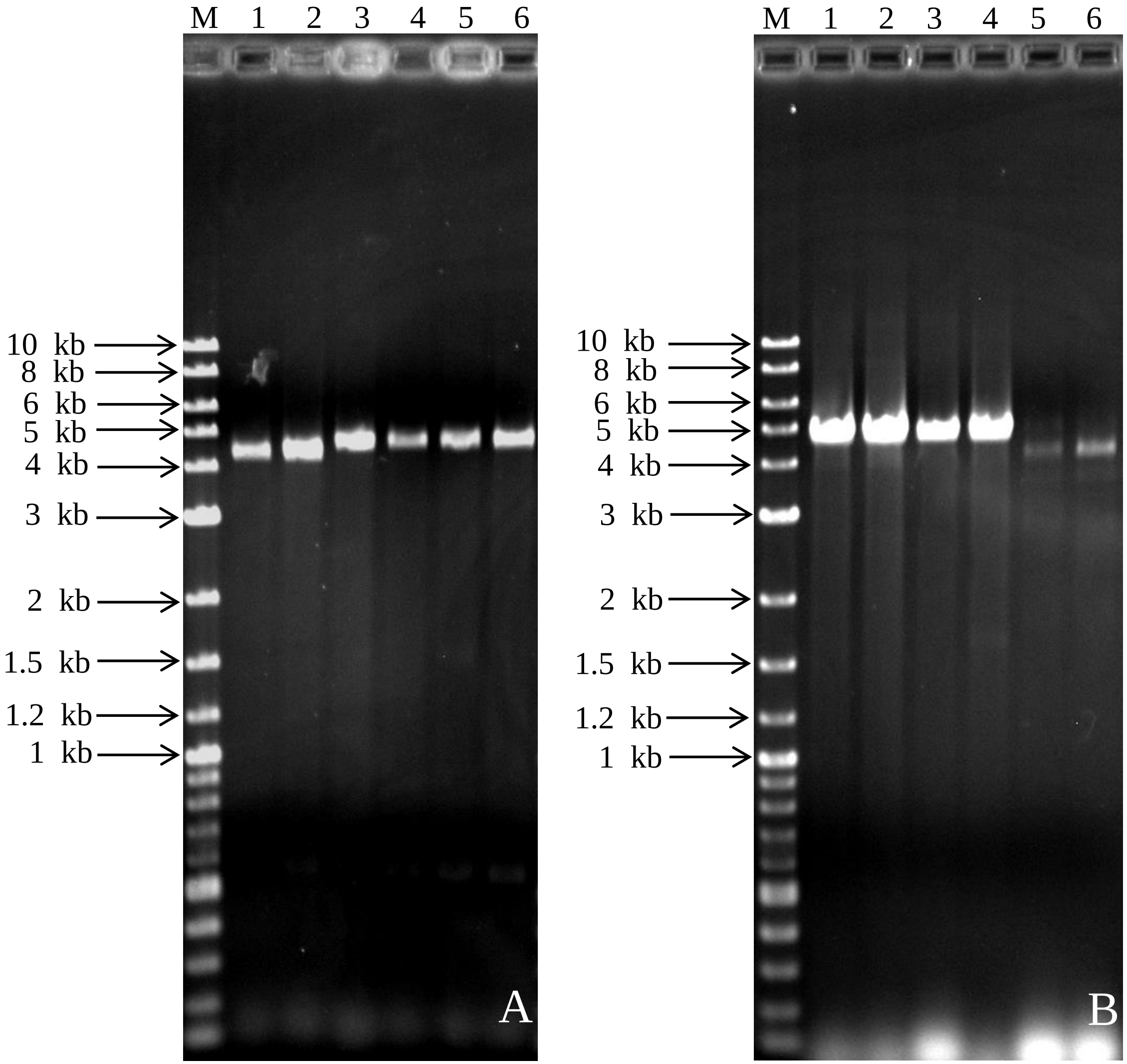

Supplementary Figure S3. PCR amplification of the *SalFAD3.LA1* and *SalFAD3.LA2* genes. **A**. PCR amplification of the genomic DNA sequences of *SalFAD3.LA1* gene using the primer pair No 5 (Table S1). Lanes 1-2: 4268 bp fragment of *la*^{1} from Y1127; Lanes 3-6: 4534 bp fragment of *LA*^{1} from Y1035. **B**. PCR amplification of the genomic DNA sequences of *SalFAD3.LA2* gene using the primer pair No 6 (Table S1). Lanes 1-4: 4688 bp fragment of *la*^{2} from Y1127; Lanes 5-6: 4042 bp fragment of *LA*^{2} from Y1035.
