# Supplementary material for: Detection and Molecular Characterization of Two FAD3 Genes Controlling Linolenic Acid Content and Development of Allele-Specific Markers in Yellow Mustard (Sinapis alba)
Source: PLoS One. 2014 May 13;9(5):e97430. doi: 10.1371/journal.pone.0097430 (PMC4019595; doi:10.1371/journal.pone.0097430)
Supplement: Figure S4 — Nucleotide sequences of intron 3 and its flanking of SalFAD3.LA1 and SalFAD3.LA2 genes in yellow mustard. The sequence of exon 3 was underlined in red while that of exon 4 was lined in blue. The nucleotide sequence of the inserted fragment in exon 3 of la1 was underlined in pink. The new intron splicing site GT in the inserted fragment was indicated in green rectangle box. The nucleotides in blue rectangle box indicated the inserted fragment that remained in the CDS of exon 3 of la1. The nucleotides in red rectangle box indicated the 5 bp direct repeat (5′-AGAAC-3′). The first and the last nucleotides of intron 3 were indicated by arrowhead and arrow, respectively. The intron 3 of LA1, la1 LA2, la2 are 647 bp, 415 bp, 530 bp and 1165 bp in length, respectively. (PDF) [file pone.0097430.s004.pdf]

```
                        *        20         *        40         *        60         *        80
Intron 3 of LA1 : GAGAATAAGCCATCGGACACACCACCAGAAC-------------------------------------------------- :  31
Intron 3 of la1 : GAGAATAAGCCATCGGACACACCACCAGAACTAGGCCTGGGCGTTCGGGGCCCCGTCGGATTCGGTTCGGTTAATTTGGAT :  81
Intron 3 of LA2 : GAGAATAAGCCATCGGACACACCACCAGAAC-------------------------------------------------- :  31
Intron 3 of la2 : GAGAATAAGCCATCGGACACACCACCAGAAC-------------------------------------------------- :  31

                        *       100         *       120         *       140         *       160
Intron 3 of LA1 : --------------------------------------------------------------------------------- :   -
Intron 3 of la1 : TTTCGGATTTCGGTATTATGTTCATAAGCCCCATTCGGTTTTTTTCTCTTATTGAATGGGTTTCGGTTCGGTTCGGTTTT :  162
Intron 3 of LA2 : --------------------------------------------------------------------------------- :   -
Intron 3 of la2 : --------------------------------------------------------------------------------- :   -

                       *       180         *       200         *       220         *       240
Intron 3 of LA1 : --------------------------------------------------------------------------------- :   -
Intron 3 of la1 : TTCCGGATTCGGTTCGGATTGGATCGGATTGTAACCAATGTTTGAAATCGTCAAAAAAATATTTTTTTAAATCATGAAAAA :  243
Intron 3 of LA2 : --------------------------------------------------------------------------------- :   -
Intron 3 of la2 : --------------------------------------------------------------------------------- :   -

                      *       260         *       280         *       300         *       320
Intron 3 of LA1 : --------------------------------------------------------------------------------- :   -
Intron 3 of la1 : ATGACAAAAAAAAACTCAAAATATATAAATTATTCACAAAACTAATTAAAAATTAGTTAATTTATCTAAACTAACTGAAAA :  324
Intron 3 of LA2 : --------------------------------------------------------------------------------- :   -
Intron 3 of la2 : --------------------------------------------------------------------------------- :   -

                     *       340         *       360         *       380         *       400
Intron 3 of LA1 : --------------------------------------------------------------------------------- :   -
Intron 3 of la1 : GTATTAAAAATAATAAATAAAATAAATTATAAAATATTTAGAATATTATAAAATATCTGAATTACTTTAATATATATAAAA :  405
Intron 3 of LA2 : --------------------------------------------------------------------------------- :   -
Intron 3 of la2 : --------------------------------------------------------------------------------- :   -

                    *       420         *       440         *       460         *       480
Intron 3 of LA1 : --------------------------------------------------------------------------------- :   -
Intron 3 of la1 : ATATTAGCATATTTAACTAACTTTGGATGTCTTCGGATCCTAGTTTGGATTTCGGTTCGGTTCAAGTTATAACCAAACCCC :  486
Intron 3 of LA2 : --------------------------------------------------------------------------------- :   -
Intron 3 of la2 : --------------------------------------------------------------------------------- :   -

                   *       500         *       520         *       540         *       560
Intron 3 of LA1 : --------------------------------------------------------------------------------- :   -
Intron 3 of la1 : AAAGTATTAAGATCACAAGTCCCGTTCGGATATTTTTGTATAACGGTTTGGATCCGGTTTCGGTTTTTCGGTTCGGGTTT :  567
Intron 3 of LA2 : --------------------------------------------------------------------------------- :   -
Intron 3 of la2 : --------------------------------------------------------------------------------- :   -

                  *       580         *       600         *       620         *       640
Intron 3 of LA1 : ------------------------------------------------CATGGCCATGTTGAAAACGACGAGTCTTGGGTT :  64
Intron 3 of la1 : AGGTAAGTAATTCGGGTTGGGGTAGAAACGCCCAGGCCTAACCAGAACCATGGCCATGTTGAAAACGACGAGTCTTGTGTT :  648
Intron 3 of LA2 : ------------------------------------------------CATGGCCATGTTGAAAACGACGAGTCTTGGGTT :  64
Intron 3 of la2 : ------------------------------------------------CATGGCCATGTTGAAAACGACGAGTCTTGGGTT :  64

                  *       660         *       680         *       700         *       720
Intron 3 of LA1 : CCGGTAATCTTTCC--CTCTCATAGT--------------ATTTTTTTTTCCTTTTTTGAAATTATT-TCCTTTTAATTTT :  128
Intron 3 of la1 : CCGGTAATCTTTCC--CTCTCATAGT--------------ATTTTTTTTTTCTTTTTTGAAATTATT-TCCTTTTAATTTT :  712
Intron 3 of LA2 : CCGGTAATCTTTCATACTCTCATAGTTTCTCTTGACTTTTATTTATTTGTTTGTTTTCGGAACTATTCTTATGTCTATGTT :  145
Intron 3 of la2 : CCGGTAATCTTTCCTACTCTCATAGTTTCTCTTGTCTTTTATTTATTTGTTTGTTTTCGGAACTATTCTTATGTCTATGTT :  145

                  *       740         *       760         *       780         *       800         *
Intron 3 of LA1 : CTTAGGATTCTATGTATTTATTTTA---ATTAATA----------------------------TTTTTCCAG--------- :  169
Intron 3 of la1 : CTTAGGATTCTATGTATTTATTTTA---ATTAATA----------------------------TTTTTCCAG--------- :  753
Intron 3 of LA2 : CTTAGGATTCTATATGTTTATTTTATTAGTTTATG------------------------------TTTTCAG--------- :  187
Intron 3 of la2 : CTTAGGATTCGACGTGTTTATTTTATTAGTTTATGATTTTCTTAGTGGTTTATAGTAGTAACTCTTTTTCAGAAAATGAAA :  226

                      820         *       840         *       860         *       880         *
Intron 3 of LA1 : ---------TTCGAGGCTAGGACGACCATTT------GTCAGA-ACTGT----CGTTTAGCTGTAGTAA-AAAATGATTTA :  229
Intron 3 of la1 : ---------TTCGAGGCTTGGACGACCACTT------GTCAGA-ACTGT----CGTTTAGCTGTAGTAA-AAAATGATTTT :  813
Intron 3 of LA2 : ---------TTTGAGGCCAGACCGACCATTT------GTTAG--GTTGT---TTTTTCAGCTATAGTAAAAAAACAATTTG :  248
Intron 3 of la2 : AATCAATACTTTTATACTACTCCCTCCGTTTCAAAATGTTACATGTTTTAGGTTTTTCACACATATTAATAAAACATATTA :  307
```

```
                     900 * 920 * 940 * 960 *
Intron 3 of LA^1 : ------------AAGTG---------------------------------------------------------------- :  234
Intron 3 of la^1 : ------------AAGTG---------------------------------------------------------------- :  818
Intron 3 of LA^2 : -----------CAAGTGTA--------------------------ATAGTTC---ACCAGT-----------------GAT :  272
Intron 3 of la^2 : AATCTTAACATTAAATGTATAATTTTTTGTGATTAACTATTTCCCATAACTTTTAACCAATCAAAATTCAATAAGTACAAT :  388

                     980 * 1000 * 1020 * 1040 *
Intron 3 of LA^1 : --------------GTTTATAGT------------ACT------------------------------------------- :  246
Intron 3 of la^1 : --------------GTTTATAGT------------ACT------------------------------------------- :  830
Intron 3 of LA^2 : TGATTATCTTAGTGGTTTACAGT--------AGTAACT-----------CTTTCAAAAAATGAAAAAT----------CAA :  324
Intron 3 of la^2 : TAATTTTCTTGA-AGTTTACAATTTATCATTATTAACTACTAAAAATTGCATTGAAAAAGTAAAAAATGTAACTTTGACAA :  468

                     1060 * 1080 * 1100 * 1120 *
Intron 3 of LA^1 : -------------------------------------------GTAGTTAACTTTAACAACGGACCACTTATATTCGACGT :  284
Intron 3 of la^1 : -------------------------------------------GTAGTTAACTTTAACAACGGACCACTTATATTCGACGT :  868
Intron 3 of LA^2 : TACTTTT---------AT---------------------ACTAGTACCTAACTTTAACGATGGACCACTTATATTCGAT-C :  374
Intron 3 of la^2 : AAATTTTTGCTAGAAAATGGATTATTTAGAAACGGAGGGAGTAGTACCTAACTTTAACGATGGACCACTTATATTCGAT-C :  548

                     1140 * 1160 * 1180 * 1200 *
Intron 3 of LA^1 : CATTGGCATAAAATGATTCTCCTCGATATTCGTTTACTTTTCTTAGTATTTTTCT------GTTTTGGAGCTTTCAT---- :  355
Intron 3 of la^1 : CATTGGCATAAAATGATTCTCCTCGATATTCGTTTACTTTTCTTAGTATTTTTCT------GTTTTGGAGCTTTCAT---- :  939
Intron 3 of LA^2 : CATTAGCATAAAATGATTCTCCTCGAAATCCGTTTACTTTCTTCATTATTTTTTTCAAACAGTTTTGGCGTTTTGTTTGCA :  455
Intron 3 of la^2 : CATTAGCATAAAATGATTCTCCTCGAAATCCGTTTACTTTCTTCATTATTTTTATCAAACAGTTTTGGCGTTTTGTCTGCA :  629

                     1220 * 1240 * 1260 * 1280 *
Intron 3 of LA^1 : ------AGAACTAATAA-AAAGAAAAAACTTATAAA-------CACACCACATGCAGTTAATTAATTTCAACATATAATAT :  422
Intron 3 of la^1 : ------AGAACTAATAA-AAAGAAAAAACTTATAAACATTAAACACACCACATGCAGTTAGTTAATTCCAACATATAATAT : 1013
Intron 3 of LA^2 : ATCTTGAAAACTATTATGAAATTGAAAACTTATA---------CACATCACATGCAATGAATTAATACGAATATATAACCA :  527
Intron 3 of la^2 : ATCTTGAAAACTATTAT-AAATTGAAAACTTATA---------CACATCACATGCAATGAATTAATACGAATATATAACCA :  700

                     1300 * 1320 * 1340 * 1360 * 1380
Intron 3 of LA^1 : TAACTATAA-TTTTAAAT-ATTACTCATTCC-------GTTTCGCAAAGAGTGTCGTTTTAGA------------------ :  476
Intron 3 of la^1 : TAACTATAA-TGTTAAAT--------------------------------------------------------------- : 1030
Intron 3 of LA^2 : TAAGGACAAATTTTCAATGAATATTTAATA--------------------------------------------------- :  557
Intron 3 of la^2 : GAAGGACAAATTTTCAATGAATATTTAATACTCCCTCTGTTTTATAATAAGTGTCATTTTAGATTTATGCACACGGATTAA :  781

                     * 1400 * 1420 * 1440 * 1460
Intron 3 of LA^1 : -GAAATTTTTCATTATA---------------------------------------------------------------- :  492
Intron 3 of la^1 : --------------------------------------------------------------------------------- :    -
Intron 3 of LA^2 : --------------------------------------------------------------------------------- :    -
Intron 3 of la^2 : GAAAACATTTAATTTTACATATTTTCTATATAAAAACATCATTTCCCATACACCTAACTATATTTCAACCATAGAAAATAA :  862

                     * 1480 * 1500 * 1520 * 1540
Intron 3 of LA^1 : --------------------------------------------------------------------------------- :    -
Intron 3 of la^1 : --------------------------------------------------------------------------------- :    -
Intron 3 of LA^2 : --------------------------------------------------------------------------------- :    -
Intron 3 of la^2 : AATTGGAAAATAAAATTCTTAAATTTTGTATTGAAAAACGAAAACGACACTTATTTCGTAACAAAAAATTTCCTCTAAAAC :  943

                     * 1560 * 1580 * 1600 * 1620
Intron 3 of LA^1 : -----------------------------AAATAAATGTCGTTTTATGATTTCAATGC----------------------- :  521
Intron 3 of la^1 : ------------------------------ATTAA---------------------------------------------- : 1035
Intron 3 of LA^2 : ---------------------------GTAAGTAA---------------------------------------------- :  565
Intron 3 of la^2 : GACACTCTTTGTGAAACGGAGGGAGTAGTAAGTAAGTACTATTCTCTCCGTTTTATATTATTTGTCGTTGTAGAAAAAAAA : 1024

                     * 1640 * 1660 * 1680 * 1700
Intron 3 of LA^1 : ATAATTTATTGATTTTA------------TTTTTAGTTTAATTTTCTATTGGTTGAAATATGATTAGGTATATGGGTAATG :  590
Intron 3 of la^1 : --------------------------------------------------------------------------------- :    -
Intron 3 of LA^2 : --------------------------------------------------------------------------------- :    -
Intron 3 of la^2 : ATTGTTTATCGTTTTCAATGCAAAATAATTTTTCACTTTATTTTTTTATTGATTGAAATATGATTAAATGTATAGATAATT : 1105

                     * 1720 * 1740 * 1760 * 1780
Intron 3 of LA^1 : ATGTTTTTCTATAGAAAATATGCAAAATTAAATGTTTTCTTAATCTGTGTGCAAAAAGCTAAAACGACATTTGTTATGAAA :  671
Intron 3 of la^1 : --------------------------------------------------------------------------------- :    -
Intron 3 of LA^2 : --------------------------------------------------------------------------------- :    -
Intron 3 of la^2 : ATGTTTTTATTTAGAAAATATATAAAATTATATATTTTCTTAATATGTATGCATAAATCTATAATGACGAGTAATATAAAA : 1186
```

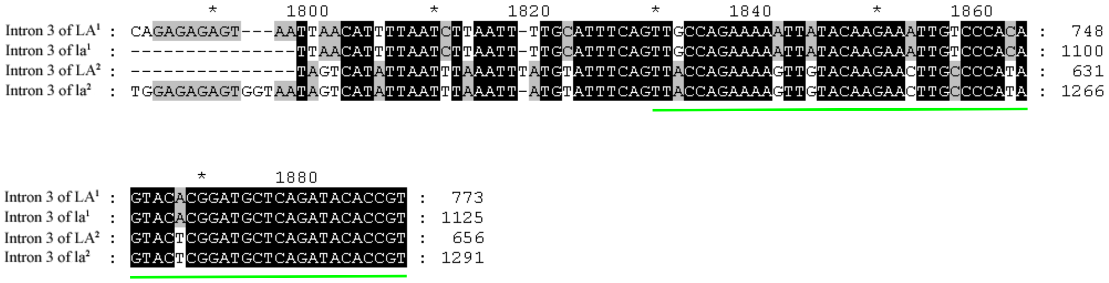

Supplementary Figure S4. Nucleotide sequences alignment of intron 3 of *SalFAD3.LA1* and *SalFAD3.LA2* genes in yellow mustard. The sequence of exon 3 was underlined in red while that of exon 4 was underlined in green. The nucleotide sequence of inserted fragment in exon 3 of la^1 was from position 31 to 615. The new intron splicing site GT in the inserted fragment in exon 3 of *la^1* was underlined in black. The nucleotides in blue rectangle indicated the inserted fragment that remained in the CDS of exon 3 of *la^1*. The nucleotides in black rectangle indicated the 5 bp direct repeat (5'-AGAAC-3').
