# Supplementary material for: Detection and Molecular Characterization of Two FAD3 Genes Controlling Linolenic Acid Content and Development of Allele-Specific Markers in Yellow Mustard (Sinapis alba)
Source: PLoS One. 2014 May 13;9(5):e97430. doi: 10.1371/journal.pone.0097430 (PMC4019595; doi:10.1371/journal.pone.0097430)
Supplement: Figure S5 — Amino acid sequences encoded by the SalFAD3.LA1 allele LA1 and the SalFAD3.LA2 allele LA2 of yellow mustard, AFJ19039.1 ( BraA.FAD3a ) and AFJ19037.1 ( BnaC.FAD3b ) of B. napus , AGH20189.1 of B. oleracea , ADJ58020.1 of B. juncea , Bra018348 of B. rapa , NP_180559.1 of A. thaliana . The amino acid sequence alignment was carried out by ClustalW2 (http://www.ebi.ac.uk/Tools/msa/clustalw2/). (PDF) [file pone.0097430.s005.pdf]

```
                      *        20         *        40         *        60         *        80
LA1        : MVVAMDQRSNVNGDS------SEERFDPSAQPPFKIGDIRAAIPKHCWVKSPLRSMSYVARDIFAVVALAVAAVYFNSWLF :  75
LA2        : MVVAMDQRSNVNGVSGG---RKEEGFDPSAQPPFKIGDIRAAIPKHCWVKSPLRSMSYVVRDIVAVAAMAMAAVYFDSWFL :  78
AFJ19039.1 : MVVAMDQRSNVNGDSGA---RKEEGFDPSEQPPFKIGDIRAAIPKHCWVKSPLRSMSYVARDIFAVAALAMAAVYFDSWFL :  78
AFJ19037.1 : MVVAMDQRSNVNGDS------KDERFDPSAQPPFKIGDIRAAIPKHCWVKSPLRSMSYVARDIFSVVALAVAAVYFDSWFF :  75
AGH20189.1 : MVVAMDQRSNVNGDS------KDERFDPSAQPPFKIGDIRAAIPKHCWVKSPLRSMSYVARDIFAVVALAVAAVYFDSWFF :  75
ADJ58020.1 : MVVAMDQRSNANGD---------ERFDPSAQPPFKIGDIRAAIPKHCWVKSPLRSMSYVARDIFAVVALAVAAVYFDSWFF :  72
Bra018348  : MVVAMDQRSNVNGDSGA---RKEEGFDPSEQPPFKIGDIRAAIPKHCWVKSPLRSMSYVARDIFAVAALAMAAVYFDSWFL :  78
NP_180559. : MVVAMDQRTNVNGDPGAGDRKKEERFDPSAQPPFKIGDIRAAIPKHCWVKSPLRSMSYVVRDIIAVAALAIAAVYVDSWFL :  81

                     *       100         *       120         *       140         *       160
LA1        : WPLYWAAQGTLFWAIFVLGHDCGHGSFSDIPLLNTVVGHILHSFILVPYHGWRISHRTHHQNHGHVENDESWVPLPEKLYK : 156
LA2        : WPLYWAAQGTLFWAIFVLGHDCGHGSFSDIPLLNSVVGHILHSFILVPYHGWRISHRTHHQNHGHVENDESWVPLPEKLYK : 159
AFJ19039.1 : WPLYWVAQGTLFWAIFVLGHDCGHGSFSDIPLLNSVVGHILHSFILVPYHGWRISHRTHHQNHGHVENDESWVPLPEKLYK : 159
AFJ19037.1 : WPLYWAAQGTLFWAIFVLGHDCGHGSFSDIPLLNTAVGHILHSFILVPYHGWRISHRTHHQNHGHVENDESWVPLPEKLYK : 156
AGH20189.1 : WPLYWAAQGTLFWAIFVLGHDCGHGSFSDIPLLNTAVGHILHSFILVPYHGWRISHRTHHQNHGHVENDESWVPLPEKLYK : 156
ADJ58020.1 : WPLYWAAQGTLFWAIFVLGHDCGHGSFSDISLLNTAVGHILHSFILVPYHGWRISHRTHHQNHGHVENDESWVPLPEKLYK : 153
Bra018348  : WPLYWVAQGTLFWAIFVLGHDCGHGSFSDIPLLNSVVGHILHSFILVPYHGWRISHRTHHQNHGHVENDESWVPLPEKLYK : 159
NP_180559. : WPLYWAAQGTLFWAIFVLGHDCGHGSFSDIPLLNSVVGHILHSFILVPYHGWRISHRTHHQNHGHVENDESWVPLPERVYK : 162

                    *       180         *       200         *       220         *       240
LA1        : KLSHSTRMLRYTVPLPMLAYPLYLWYRSPGKEGSHYNPYSSLFAPSERNLIATSTTCWSIMLATLVYLSFLVGPFTVLKVY : 237
LA2        : NLPHSTRMLRYTVPLPMLAYPIYLWYRSPGKEGSHFNPYSSLFAPSERKLIATSTTCWSIMLATLVYLSFLVGPVTVLKVY : 240
AFJ19039.1 : NLPHSTRMLRYTVPLPMLAYPIYLWYRSPGKEGSHFNPYSSLFAPSERKLIATSTTCWSIMLATLVYLSFLVGPVTVLKVY : 240
AFJ19037.1 : NLSHSTRMLRYTVPLPMLAYPLYLWYRSPGKEGSHYNPYSSLFAPSERKLIATSTTCWSIVLATLVYLSFLVGPVTVLKVY : 237
AGH20189.1 : NLSHSTRMLRYTVPLPMLAYPLYLWYRSPGKEGSHYNPYSSLFAPSERKLIATSTTCWSIVLATLVYLSFLVGPVTVLKVY : 237
ADJ58020.1 : NLSHSTRMLRYTVPLPMLAYPLYLWYRSPGKEGSHYNPYSSLFAPSERKLIATSTTCWSIMLATLVYLSFLVGPVTVLKVY : 234
Bra018348  : NLPHSTRMLRYTVPLPMLAYPIYLWYRSPGKEGSHFNPYSSLFAPSERKLIATSTTCWSIMLATLVYLSFLVGPVTVLKVY : 240
NP_180559. : KLPHSTRMLRYTVPLPMLAYPLYLCYRSPGKEGSHFNPYSSLFAPSERKLIATSTTCWSIMFVSLIALSFVFGPLAVLKVY : 243

                   *       260         *       280         *       300         *       320
LA1        : GVPYIIFVMWLDAVTYLHHHGHDDKLPWYRGKEWSYLRGGLTTVDRDYGIFNNIHHDIGTHVIHHLFPQIPHYHLVDATKA : 318
LA2        : GVPYIIFVMWLDAVTYLHHHGHDGKLPWYRGKEWSYLRGGLTTIDRDYGIFNNIHHDIGTHVIHHLFPQIPHYHLVDATKA : 321
AFJ19039.1 : GVPYIIFVMWLDAVTYLHHHGHDEKLPWYRGKEWSYLRGGLTTIDRDYGIFNNIHHDIGTHVIHHLFPQIPHYHLVDATRA : 321
AFJ19037.1 : GVPYIIFVMWLDAVTYLHHHGHDDKLPWYRGKEWSYLRGGLTTVDRDYGIFNNIHHDIGTHVIHHLFPQIPHYHLVDATKA : 318
AGH20189.1 : GVPYIIFVMWLDAVTYLHHHGHDDKLPWYRGKEWSYLRGGLTTVDRDYGIFNNIHHDIGTHVIHHLFPQIPHYHLVDATKA : 318
ADJ58020.1 : GVPYIIFVMWLDAVTYLHHHGHDDKLPWYRGKEWSYLRGGLTTIDRDYGIFNNIHHDIGTHVIHHLFPQIPHYHLVDATKS : 315
Bra018348  : GVPYIIFVMWLDAVTYLHHHGHDEKLPWYRGKEWSYLRGGLTTIDRDYGIFNNIHHDIGTHVIHHLFPQIPHYHLVDATRA : 321
NP_180559. : GVPYIIFVMWLDAVTYLHHHGHDEKLPWYRGKEWSYLRGGLTTIDRDYGIFNNIHHDIGTHVIHHLFPQIPHYHLVDATKA : 324

                  *       340         *       360         *       380
LA1        : AKHVLGRYYREPKTSGAIPIHLVESLVASIKKDHYVSDTGDIVFYETDPDLYVYASDKSKIN : 380
LA2        : AKHVLGRYYREPKTSGAIPIHLVESLVASIKKDHYVSDTGDIVFYETDPDLYVYASDKSKIN : 383
AFJ19039.1 : AKHVLGRYYREPKTSGAIPIHLVESLVASIKKDHYVSDTGDIVFYETDPDLYVYASDKSKIN : 383
AFJ19037.1 : AKHVLGRYYREPKTSGAIPIHLVESLVASIKKDHYVSDTGDIVFYETDPDLYVYASDKSKIN : 380
AGH20189.1 : AKHVLGRYYREPKTSGAIPTHLVESLVASIKKDHYVSDTGDIVFYETDPDLYVYASDKSKIN : 380
ADJ58020.1 : AKHVLGRYYREPKTSGAIPIHLVESLVASIKKDHYVSDTGDIVFYETDPDLYVYASDKSKIN : 377
Bra018348  : AKHVLGRYYREPKTSGAIPIHLVESLVASIKKDHYVSDTGDIVFYETDPDLYVYASDKSKIN : 383
NP_180559. : AKHVLGRYYREPKTSGAIPIHLVESLVASIKKDHYVSDTGDIVFYETDPDLYVYASDKSKIN : 386
```

Supplementary Figure S5. Amino acid sequences encoded by the *SalFAD3.LA1* allele LA^1 and the *SalFAD3.LA2* allele LA^2 of yellow mustard, AFJ19039.1 (*BraA.FAD3a*) and AFJ19037.1 (*BnaC.FAD3b*) of *B. napus*, AGH20189.1 of *B. olerarea*, ADJ58020.1 of *B. juncea*, Bra018348 of *B. rapa*, NP_180559 of *A. thaliana*. The amino acid sequence alignment was carried out by ClustalW2 (http://www.ebi.ac.uk/Tools/msa/clustalw2/).
