# Supplementary material for: Detection and Molecular Characterization of Two FAD3 Genes Controlling Linolenic Acid Content and Development of Allele-Specific Markers in Yellow Mustard (Sinapis alba)
Source: PLoS One. 2014 May 13;9(5):e97430. doi: 10.1371/journal.pone.0097430 (PMC4019595; doi:10.1371/journal.pone.0097430)
Supplement: Table S1 — Primers used in this study. (PDF) [file pone.0097430.s006.pdf]

Supplementary Table S1 Primers used in this study.

No	Primer	Sequence (5'-3')	Purpose
1	FAD3 F	ATGGTTGT**YG**CTATGGACC	To clone the coding region of the *FAD3* gene
	FAD3 R	TTAATTGATTTTGGATTTGTCAGAAGC	
2	GSPA1	AATCGCCGCTCTTATATCTCCGATCTT	To get the 5' flanking sequence
	GSPA2	TCTTCCGAGGAATCTCCGTTCACATTG	
3	GSPB1	TCAAACCCTTCTTCCTTCCGGCCACCG	
	GSPB2	CGGCCACCGGAGACTCCGTTCACATTG	
4	DGSP1	ACATTCATCACGATATTGGAACTCACG	To get the 3' flanking sequence
	DGSP2	ACACTGGTGATATTGTCTTCTACGAGA	
5	AP F	GCGATGGTTACTTATACTCA	To get the genomic DNA sequences of LA^1 and la^1
	AW R	AATAGAGCTAGGAAGATAGG	
6	BP F	CTGGTATCTTCGAATCTCAT	To get the genomic DNA sequences of LA^2 and la^2
	BW R	CGTGACACATTGTTGTTGTA	
7	EX1-2 F	GAGAATAAGCCATCGGACAC	To sequence the whole gene
	EX1-2 R	ACGAGGATGAATGAATGAAG	
8	EX2-3 F	TCTGCTGAACAGTGTGGTGG	
	EX2-3 R	CATGGTTCTGGTGGTGTGTC	
9	EX3-4 F	AGGAGAATAAGCCATCGGAC	
	EX3-4 R	GGACGGTGTATCTGAGCATC	
10	EX4-7 F	TACTCGGATGCTCAGATACA	
	EX4-7 R	CGTGATGGATGTTGTTGAAG	
11	EX5-U F	CAACTTCAACTACTTGCTGG	
	EX5-U R	AATAGAGCTAGGAAGATAGG	
12	SalFAD3 F	GCCATGTTGAAAACGACGAG	To generate *FAD3* allele-specific markers
	SalFAD3 R	ACAGTGTATCTGAGCATCCG	
